# Altering the mRNA-1273 dosing interval impacts the kinetics, quality, and magnitude of immune responses in mice

**DOI:** 10.3389/fimmu.2022.948335

**Published:** 2022-11-08

**Authors:** Dario Garcia-Dominguez, Carole Henry, LingZhi Ma, Hardik Jani, Nicholas J. Amato, Taylor Manning, Alec Freyn, Heather Davis, Chiaowen Joyce Hsiao, Mengying Li, Hillary Koch, Sayda Elbashir, Anthony DiPiazza, Andrea Carfi, Darin Edwards, Kapil Bahl

**Affiliations:** Moderna, Inc., Cambridge, MA, United States

**Keywords:** SARS-CoV-2, mRNA-1273, COVID-19, dosing interval, dosing regimen

## Abstract

For a vaccine to achieve durable immunity and optimal efficacy, many require a multi-dose primary vaccination schedule that acts to first “prime” naive immune systems and then “boost” initial immune responses by repeated immunizations (ie, prime-boost regimens). In the context of the global coronavirus disease 2019 (COVID-19) pandemic caused by severe acute respiratory syndrome coronavirus 2 (SARS-CoV-2), 2-dose primary vaccination regimens were often selected with short intervals between doses to provide rapid protection while still inducing robust immunity. However, emerging post-authorization evidence has suggested that longer intervals between doses 1 and 2 for SARS-CoV-2 vaccines may positively impact robustness and durability of immune responses. Here, the dosing interval for mRNA-1273, a messenger RNA based SARS-CoV-2 vaccine administered on a 2-dose primary schedule with 4 weeks between doses, was evaluated in mice by varying the dose interval between 1 and 8 weeks and examining immune responses through 24 weeks after dose 2. A dosing interval of 6 to 8 weeks generated the highest level of antigen-specific serum immunoglobulin G binding antibody titers. Differences in binding antibody titers between mRNA-1273 1 µg and 10 µg decreased over time for dosing intervals of ≥4 weeks, suggesting a potential dose-sparing effect. Longer intervals (≥4 weeks) also increased antibody-dependent cellular cytotoxicity activity and numbers of antibody-secreting cells (including long-lived plasma cells) after the second dose. An interval of 6 to 8 weeks elicited the strongest CD8+ T-cell responses, while an interval of 3 weeks elicited the strongest CD4+ T-cell response. Overall, these results suggest that in a non-pandemic setting, a longer interval (≥6 weeks) between the doses of the primary series for mRNA-1273 may induce more durable immune responses.

## Introduction

Vaccination aims to provide protection against infection and/or disease when an individual is subsequently exposed to the causative pathogen (1). Achievement of vaccine-mediated protection relies on the ability of a vaccine to elicit potent and durable immune responses to a specific pathogen (1); this can be affected by a multitude of vaccine-specific variables, including the immunizing antigen, antigen-delivery system (ie, nucleic acid, viral vector, recombinant protein, or inactivated pathogen), immunization route, adjuvant, dosing level, and regimen, as well as characteristics of the vaccine recipient, including prior infection, race/ethnicity, age, and gender (2, 3).

Vaccine-mediated protection often correlates with the magnitude of antibody responses (1, 4, 5), with T cells playing a potentially equally important role, particularly in the context of waning antibody responses (6). Upon pathogen exposure after vaccination, antigen-specific B and T cells increase in frequency and differentiate into antigen-specific memory cells (ie, immunological memory), enabling the immune system to respond quickly and robustly to a re-encountered antigen (1, 7–9).

To establish immunity, vaccines are typically administered on a multi-dose primary vaccination schedule, first to “prime” a naive immune system and subsequently to “boost” immune responses through repeated administrations (ie, prime-boost regimens) (10, 11). However, defining the ideal interval between the doses of the primary vaccination series remains difficult and is not well understood. Current recommendations from the World Health Organization indicate that routine immunizations among children worldwide typically have a minimum 4-week interval between doses 1 and 2 (12); however, the exact interval can vary by vaccine type, antigen, regional location, and population age, among other factors. Understanding how the dosing interval impacts vaccine-elicited immune responses is thus of significant importance for ensuring robust and durable vaccine-mediated protection.

Since the emergence of severe acute respiratory syndrome coronavirus 2 (SARS-CoV-2) and the ensuing coronavirus disease 2019 (COVID-19) pandemic, several SARS-CoV-2 vaccines have been developed, including mRNA-1273 (SPIKEVAX; Moderna, Inc., Cambridge, MA, USA), an mRNA-based COVID-19 vaccine encoding for the SARS-CoV-2 spike protein (13). In the pivotal phase 3 clinical trial (NCT04470427), mRNA-1273 100 µg administered intramuscularly with a 4-week interval between dose 1 and 2 resulted in 93.2% efficacy against disease (14). However, emerging studies on other 2-dose SARS-CoV-2 vaccines have indicated that extending the dose interval beyond the standard 4-week schedule improved antibody and B-cell responses as well as vaccine efficacy and effectiveness, but reduced interferon γ (IFNγ)-producing T-cell responses (15–19).

We therefore evaluated how the interval between dose 1 and dose 2 of mRNA-1273 affects vaccine-elicited immunogenicity in mice, assessing multiple aspects of the kinetics, magnitude, and durability of mRNA-1273–induced immune responses across dosing intervals (from 1-8 weeks), including characterizing antigen-specific antibody and T-cell responses as well as long-term memory cell maintenance.

## Materials and methods

### Mice

**Figure 1:**
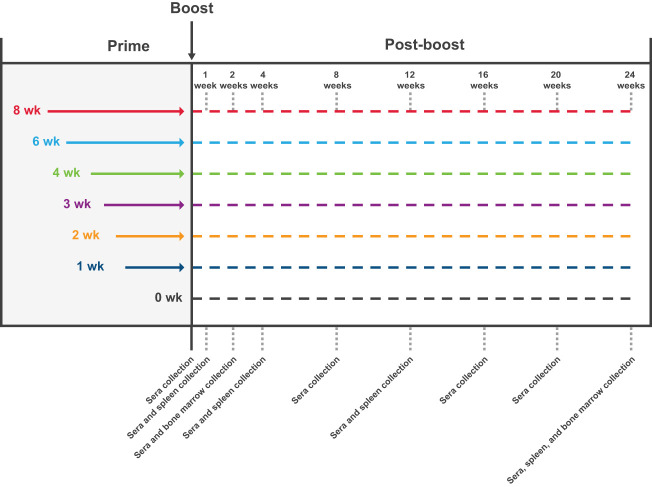
Study Design. The impact of the dosing interval on mRNA-1273 vaccine immunogenicity was evaluated by administering 2 doses of mRNA-1273 (1 µg or 10 µg) to mice on a schedule of 1-, 2-, 3-, 4-, 6-, or 8-week intervals between doses; a prime-only group (only administered first dose of mRNA-1273 at time of dose 2) was also evaluated for comparison purposes. Sera were collected from mice (n=8-10 per group) before dose 2 and 1, 2, 4, 8, 12, 16, 20, and 24 weeks following dose 2. Spleens were collected 1, 4, 12, and 24 weeks after dose 2 from a subgroup of mice (n=8-10 per group) administered mRNA-1273 10 µg; bone marrow samples were collected 2 and 24 weeks after dose 2 from the same subgroup of mice. mRNA, messenger RNA.

Specific pathogen-free, 6- to 8-week-old BALB/c mice were purchased from Charles River Laboratories and housed in microisolator cages in a BSL-2 facility with sterile water and food provided ad libitum. Animal experiments were carried out in compliance with approval from the Institutional Animal Care and Use Committee of Moderna, Inc. Mice were immunized with mRNA-1273 1 µg or 10 µg (preclinical batch [non-GMP]) diluted in phosphate-buffered saline (PBS) 50 µL *via* intramuscular injection into the same hind leg for both dose 1 and dose 2. Mice (n=8-10 mice per group) were immunized with 2 doses of mRNA-1273 (1 µg or 10 µg) at varying dosing intervals (1-, 2-, 3-, 4-, 6-, or 8-week intervals between doses; **Figure 1**); for the purposes of comparing to a single dose regimen, an additional group of mice received only a single mRNA-1273 immunization at the time of dose 2 (prime-only group). For immunogenicity assessments, samples were collected as detailed in **Figure 1**.

### Preclinical mRNA and lipid nanoparticle production

A sequence-optimized mRNA encoding the prefusion-stabilized SARS-CoV-2 spike protein with 2 proline mutations (S-2P) was synthesized *in vitro* as previously described (20). The mRNA then underwent oligo-dT affinity purification, buffer exchange by tangential flow filtration into sodium acetate (pH 5.0), sterile filtration, and stored at −20°C until use. As described previously (21), mRNA was lipid nanoparticle (LNP) encapsulated through a modified ethanol-drop nanoprecipitation process. Ionizable, structural, helper, and polyethylene glycol (PEG) lipids were mixed at a 2.5:1 ratio with mRNA (lipids:mRNA) in acetate buffer (pH 5.0). The drug product was not intended for clinical use; the product underwent analytical characterization (ie, mRNA purity, double-stranded RNA content, particle size and polydispersity determination, encapsulation, osmolality, pH, endotoxin, and bioburden) and was deemed acceptable for *in vivo* study.

### S-2P–specific enzyme-linked immunosorbent assay

Microtiter plates were coated with S-2P protein 1 µg/mL (GenScript), corresponding to the spike protein of the Wuhan-Hu-1 virus stabilized with 2 proline mutations, and incubated overnight at 4°C. Plates were then washed 4 times with PBS/0.05% Tween-20 and blocked for 1.5 hours at 37°C using SuperBlock (Thermo). After washing, 5-fold serial dilutions of mouse serum were added (assay diluent: TBS + 5% BSA + 0.05% Tween-20 [Boston Bioproducts]) and incubated for 2 hours at 37°C. Plates were washed and then horseradish peroxidase-conjugated goat anti-mouse immunoglobulin G (IgG) (Southern Biotech) was added at a 1:30,000 dilution in assay diluent. After incubation for 1 hour at 37°C, plates were washed and bound antibody was detected with a 3,3’,5,5’-tetramethylbenzidine (TMB) substrate (Thermo). After a 10-minute incubation at room temperature, TMB stop solution (Invitrogen) was added to stop the reaction and absorbance at 450 nm was measured. Titers were determined using a 4-parameter logistic curve fit in Prism v.8 (GraphPad 112 Software, Inc.), defined as the reciprocal dilution at approximately optical density 450 = 1 (normalized to a mouse standard on each plate).

### Anti-polyethylene glycol enzyme-linked immunosorbent assay

Carboxy-modified latex beads (Molecular Probes, Life Technologies) were aliquoted to 100 μL per tube and washed 3 times with 50 mM MES 1 mM EDTA (pH 6.0). Beads were coupled with 1-ethyl-3-(3-dimethylaminopropyl) carbodiimide HCl 5 mg (Thermo Fisher Scientific) on a plate shaker at maximum rpm for 15 minutes at room temperature. Coupled beads were incubated with PEG2K-DMG 400 µg in PBS for 2 hours (at room temperature shaking at 800 rpm), washed 2 times with PBS, and resuspended in PBS + 2% BSA for either direct usage or storage at 4°C. PEG-coupled beads were incubated with serum samples diluted 1:100 at room temperature for 45 minutes. Beads were then washed and incubated with either anti-mouse IgM APC (1:1000; clone Il-41; BD Pharmingen) or anti-mouse IgG Alexa Fluor 488 (1:1000; polyclonal; Abcam) for 30 minutes in the dark. After washing, beads were resuspended in PBS 100 µL + 2% BSA for analysis on a ThermoFisher Scientific Attune NXT. Forward scatter and side scatter gatings were adjusted so that the field captured the 10-µm PEG-coupled carboxy-modified latex beads; 60 µL of the sample was collected at a speed of 200 µL/s. Bead population was gated around in FlowJo 10.8, eliminating debris, and median fluorescence intensity for either APC or FITC was applied to appropriate serum samples or controls. Antibody levels for respective IgM and IgG samples were quantified using a standard curve obtained with a monoclonal mouse anti-PEG IgM (AGP4-PABM-A; Academia Sinica, Taipei, Taiwan) or a monoclonal mouse anti-PEG IgG (3.3-PABG-A; Academia Sinica, Taipei, Taiwan).

### B-cell ELISpot

Ninety-six well plates were incubated overnight at 4°C with SARS-CoV-2 S-2P protein 2 µg /mL (GenScript). On the next day, plates were washed 3 times with PBS and blocked for 2 hours at 37°C with RPMI complete medium (RPMI + 10% fetal calf serum + 1% penicillin/streptomycin + 1% HEPES + 1% L-Glutamine). Freshly isolated splenocytes or bone marrow cells were washed 3 times then resuspended in RPMI complete medium, added to each plate, and serially diluted 2-fold down the plate. After overnight incubation at 37°C, plates were washed extensively with PBS + 0.05% Tween-20 and antibody-secreting cells (ASCs) were detected with biotinylated anti-mouse IgG (1:10,000, Southern Biotech; 1030-08), followed by streptavidin-alkaline phosphatase (1:500, Southern Biotech; 7105-04), and developed with nitroblue tetrazolium–5-bromo-4-chloro-3-indolylphosphate (Thermo Scientific). Plates were imaged (Cellular Technologies) and spots were manually counted to determine the number of ASCs.

### T-cell assessments by intracellular cytokine staining

A gentleMACS tissue dissociator (Miltenyi Biotec) was used to generate mononuclear single-cell suspensions from BALB/c mouse whole spleens. Following tissue dissociation, cells were sieved through a 70-µm filter. Cells from each mouse were resuspended in R10 media (RPMI 1640 medium supplemented with L-glutamine, penicillin/streptomycin, and 10% HI−fetal bovine serum [FBS]) and incubated at 37°C for 6 hours with protein transport inhibitors GolgiStop and GolgiPlug (BD Biosciences) and 1 µg/mL spike glycoprotein peptide pools (JPT; PM−WCPV-S-1; divided into peptide pools, S1 and S2), 1 µg/mL spike RBD peptide pool (JPT; PM-WCPV-S-RBD), or 1 µg/mL spike NTD peptide pool (JPT; custom order). All pools were derived from the Wuhan-Hu-1 strain and contained peptides of 15 amino acids in length overlapped by 11 amino acids (70% purity). Control cells were incubated with an equivalent concentration of DMSO as contained in the peptide pools. Cells were washed with PBS then stained with LIVE/DEAD Fixable Aqua Dead Cell Stain (Invitrogen) for 20 minutes at room temperature. Cells were subsequently washed with FC stain buffer (PBS supplemented with 3% HI-FBS and 0.05% sodium azide) and resuspended in Becton, Dickinson and Company (BD) Fc Block (clone 2.4G2) for 5 minutes at room temperature. Staining was performed at 4°C for 30 minutes with a surface stain cocktail of the following antibodies: CD4 APC (Biolegend; 100412, clone GK1.5), CD8 Alexa Fluor 700 (Biolegend; 126618, clone YTS156.7.7), and CD44 BV421 (BD; 563970, clone IM7). After this step, cells were washed with FC buffer and then fixed and permeabilized using the BD Cytofix/Cytoperm kit according to the manufacturer’s instructions. Cells were washed with permeabilized and wash solution and then intracellular staining was performed at 4°C for 30 minutes using the following cocktail of antibodies in 1X permeabilized and wash solution: IFNγ APC-Cy7 (Biolegend; 505850, clone XMG1.2), tumor necrosis factor α (TNFα) PE-Dazzle594 (Biolegend; 506345, clone MP6-XT22), interleukin-2 (IL-2) BV711 (Biolegend; 503837, clone JES6-5H4), IL-4 PE-Cy7 (Biolegend; 504117, clone 11B11), IL-5 PE (Biolegend; 504303, clone TRFK5), IL-9 PerCP-Cy5.5 (Biolegend; 514112, clone RM9A4), IL-10 BV605 (Biolegend; 505031, clone JES5-16E3), and IL-13 Alexa Fluor 488 (ThermoFisher; 53-7133-82, clone eBio13A). Cells were then washed with permeabilized and wash solution, filtered through a 96-well plate 30-µm filter (Pall), and resuspended in FC stain buffer prior to running on a LSR Fortessa flow cytometer (BD). Analysis was done using FlowJo software (version 10.7.1). Background cytokine expression in the control cells was subtracted from that measured in the peptide pools for each individual mouse.

### ADCC reporter assay

CHO-K1 cells constitutively expressing SARS-CoV-2 S protein (GenScript) were cultured in Ham’s F-12K media containing 10% FBS and puromycin 8 μg/mL (Gibco). Cells were seeded at 1.5E4 cells/well in white-walled 96-well dishes (Corning) and incubated overnight at 37°C and 5% CO_2_. Serum was serially diluted in assay medium (RPMI 16-40 containing 4% Ultra-low IgG FBS [Gibco]), including a high-positive control and wells lacking serum as a negative control. Media was aspirated from the wells and 25 μL of assay medium was added to each well; 25 μL of diluted serum was then added to corresponding wells. Jurkat cells expressing murine FcγRIV with an NFAT-driven firefly luciferase reporter gene (Promega) were diluted in warm assay medium and 25 μL of the cell solution was added to each well. Plates were incubated for 6 hours at 37°C and 5% CO_2_ and then removed from the incubator to rest at room temperature for 10 to 15 minutes. Room temperature BioGlo luciferase substrate (Promega) was added at 75 μL per well and plates were read immediately on a Pherastar FS plate reader (BMG Labtech). Data were analyzed using Prism 9 (GraphPad) and were processed by subtracting the average plus 3 times the standard deviation of negative wells and normalizing to the average of positive control wells. Curves were fit to the data using the [Inhibitor] versus response – Variable slope (4 parameters) function, and area under the curve was determined and reported for each group.

### Statistical modeling and hypothesis testing

A generalized additive model (GAM) (22) was applied to enzyme-linked immunosorbent assay (ELISA) S-specific IgG antibody titers, with a dose- and dosing interval group-specific smooth nonlinear trend in days, and 9-dimensional thin plate spline basis. Animal-specific random effects were included to estimate group-specific time curves after dose 2. Two-way and 3-way interactions of dose, days after dose 2, and dosing interval were included to allow for interval-varying effects of dose levels between days following dose 2. For hypothesis testing of ELISA titers, we opted for a linear mixed effect model and included days following dose 2 as a categorical variable. The linear mixed effect model and the GAM model of ELISA included similar covariates. mgcv R package (23) was used for GAM and lme4 (24) was used for linear mixed model (described in **Supplementary Methods**).

ADCC activity was modeled using a Bayesian GAM using brms R package (25, 26) with the default weakly informative priors. A Bayesian model was selected over a frequentist model as a better test for group-specific differences while accounting for heterogeneous variances due to days after dose 2 and simultaneous accounting for data points falling below limit of detection. Dose- and dosing interval-specific non-linear terms, as well as day, dose, and interval interactions, were used to predict ADCC activity. To capture the heterogeneous variance observed across days following dose 2, residual variance was estimated to change linearly in days after dose 2. See **Supplementary Methods** for details.

To compare anti-PEG IgM and IgG levels between dosing interval groups, a linear mixed model (lme4) was used separately for IgM or IgG levels, modeling the effect of dosing interval groups and dose levels on titers. For comparisons of spike-specific ASCs and LLPCs, we transformed ASC and LLPC counts/million cells using a Box-Cox transformation (27). Generalized linear regression was used to model the transformed count by *g(counts) = β_0_ + β_1_ interval* for both spleen and bone marrow data, where g(.) denotes the Box-Cox transformation.

For comparisons of cytokine polyfunctionality and spike-specific CD4+ and CD8+ T-cell IFNγ responses, we used zero-inflated beta regression models (28). Cytokine polyfunctionality thresholded composition data were analyzed (see **Supplementary Methods** for thresholding details). A separate model was fit for each cell type, peptide, and each type of polyfunctionality (ie, a separate model was fit to predict the proportion of single expressors, dual expressors, and triple expressors). The model was constructed using default link functions for all 3 components of the zero-inflated beta distribution in the gamlss (22, 28) R package. The dosing interval, days following dose 2, and their interactions were used as linear predictors for the mean component; the dosing interval was used as the linear predictor for the scale parameter *σ.* Zero-inflated beta regression was similarly used to model spike-specific CD4+ and CD8+ T-cell IFNγ responses (see **Supplementary Methods** for details).

Statistical analyses were conducted with R version 4.1.2 (29). Statistical comparisons were conducted using the emmeans package in R (30), with multivariate *t* adjustment at alpha level of 0.05, except when noted otherwise. Residual diagnostics and goodness-of-fit criteria were examined for all models to affirm satisfactory model fit.

## Results

### S2-P–specific serum binding antibody titers

To evaluate the impact of the dosing intervals on mRNA-1273 elicited immunogenicity, mice were immunized with 2 doses of mRNA-1273 (1 µg or 10 µg) on varying dosing schedules of 1-, 2-, 3-, 4-, 6-, or 8-week intervals between doses 1 and 2; a single dose group (administered dose 1 only at time of dose 2) was also evaluated for comparison purposes (**Figure 1**). S2-P–specific serum binding IgG antibody titers were evaluated through 24 weeks after dose 2. Because mRNA vaccines induce robust and long-lasting germinal center (GC) responses (31, 32), and the formation of memory B cells and LLPCs has been associated with durable humoral immune responses (33), we assessed the persistence of serum antibodies through 24 weeks after dose 2.

**Figure 2:**
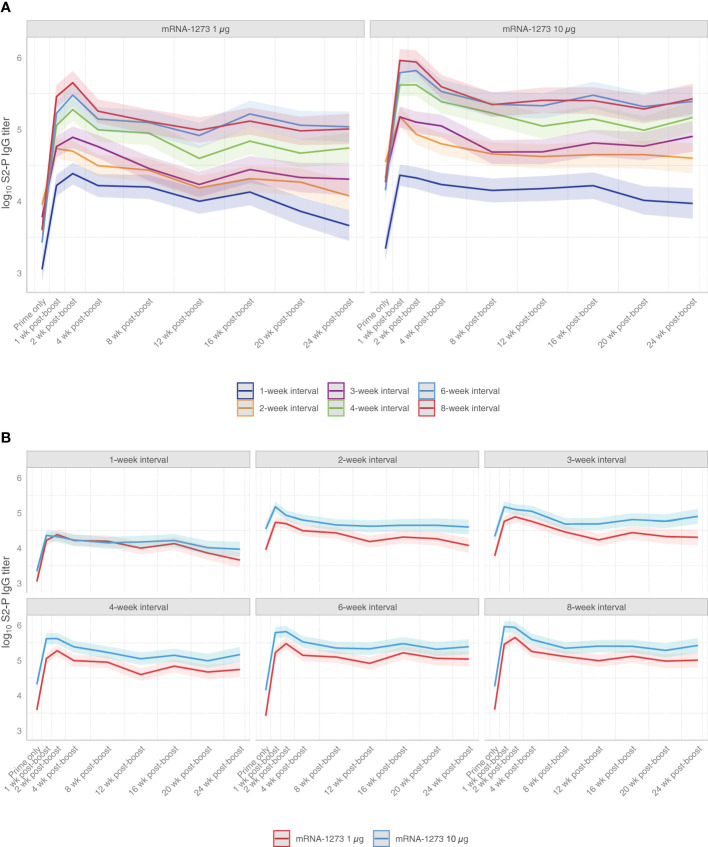
S2-P–specific Serum Binding IgG Antibody Titers. Predicted S2-P serum binding IgG antibody titers (with corresponding 95% CIs [shaded region]; based on GAM with days after dose 2 as a continuous variable [see Methods for details]) from before dose 2 through 24 weeks following dose 2 are presented according to **(A)** mRNA-1273 dosing level (1 µg or 10 µg) or by **(B)** dosing intervals (1-, 2-, 3-, 4-, 6-, or 8-week intervals between doses) with n=8-10 mice per group. Results of statistical comparisons between groups based on GAM are presented in **Table S1** and **Figure S3**. Observed data at individual animal level are shown in **Figure S1**. CI, confidence interval; GAM, generalized additive model; IgG, immunoglobulin G; mRNA, messenger RNA.

At 2 weeks following dose 2, all assessed dosing intervals of mRNA-1273 (1 µg or 10 µg dose levels) showed increased S2-P–specific antibody titers relative to titers before the second dose (**Figure 2A**; **Figure S1**), with largest fold changes observed for the 6- and 8-week intervals (**Figure S2**; **Table S1**; **Figure S3**). Increased antibody titers were also observed at 24 weeks following dose 2 for all dosing intervals except for the 2-week interval; for the 8-week interval, S2-P–specific serum-binding antibody titers at 24 weeks were approximately 30-fold and 10-fold higher relative to before dose 2 for mRNA-1273 1-µg and 10-µg dose levels, respectively (**Figure S2**). Throughout the 24-week period after dose 2, the 6- and 8-week intervals elicited the highest antibody titers, particularly in comparison to the shorter 1-, 2-, and 3-week intervals. Overall, S2-P–specific serum binding antibody titers after dose 2 were generally comparable between the 6- and 8-week intervals at all evaluated time points.

Across mRNA-1273 dose levels, S2-P–specific serum-binding antibody titers were higher at the 10-µg dose than 1-µg dose level at all time points following dose 2 for dosing intervals of ≥2 weeks (**Figure 2B**; **Figure S1**). However, differences in antibody titers between mRNA-1273 dose levels with ≥4-week intervals became progressively less observable over the 24-week study duration.

### Antibody Fc-effector responses

**Figure 3:**
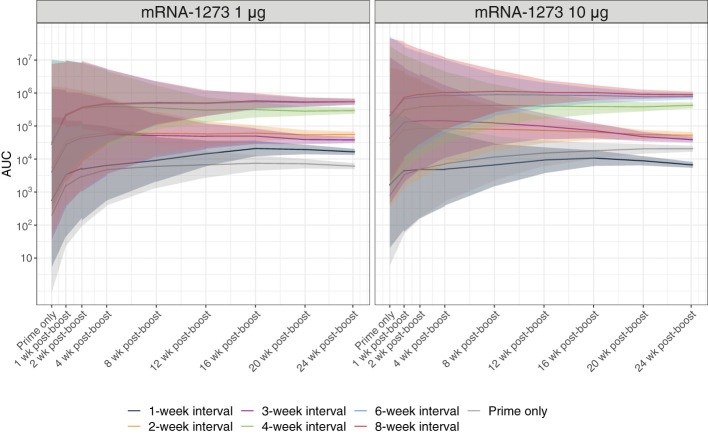
Antibody Fc-effector Function Responses. Antibody Fc-effector function was assessed using reporter cells expressing the murine FcγRIV. Predicted serum Fc-effector responses from before dose 2 through 24 weeks after dose 2 (with corresponding 95% CIs [shaded region]; based on a Bayesian GAM with days after dose 2 as a continuous variable [see Methods for details]) are presented by dosing interval (prime only, 1-, 2-, 3-, 4-, 6-, or 8-week intervals between doses) for 1-µg or 10-µg dosing levels (n=8-10 mice per group). Results of statistical comparisons between groups based on the Bayesian GAM are presented in **Table S2** and **Figure S5**. Observed data at individual animal level are shown in **Figure S4**. AUC, area under the curve; CI, confidence interval; GAM, generalized additive model; mRNA, messenger RNA.

Fc-functional antibody responses through 24 weeks after dose 2 of mRNA-1273 (1 µg or 10 µg dose levels) were evaluated using a reporter assay for ADCC activity, a regulated antibody-centric immune response that is Fc-mediated (**Figure S4**). Antibody Fc-effector responses improved with longer dosing intervals (ie, 4-, 6-, and 8-week intervals), showing significantly higher activity than ≤3-week intervals at both the 1-µg and 10-µg dose levels (adjusted *P*-value <0.001; **Figure 3**; **Table S2**; **Figure S5**). In mice immunized with mRNA-1273 10 µg, antibody responses peaked 1 week after dose 2, with longer dosing intervals showing steadily waning responses through 24 weeks after dose 2.

### Induction of S2-P–specific ASCs and LLPCs

**Figure 4:**
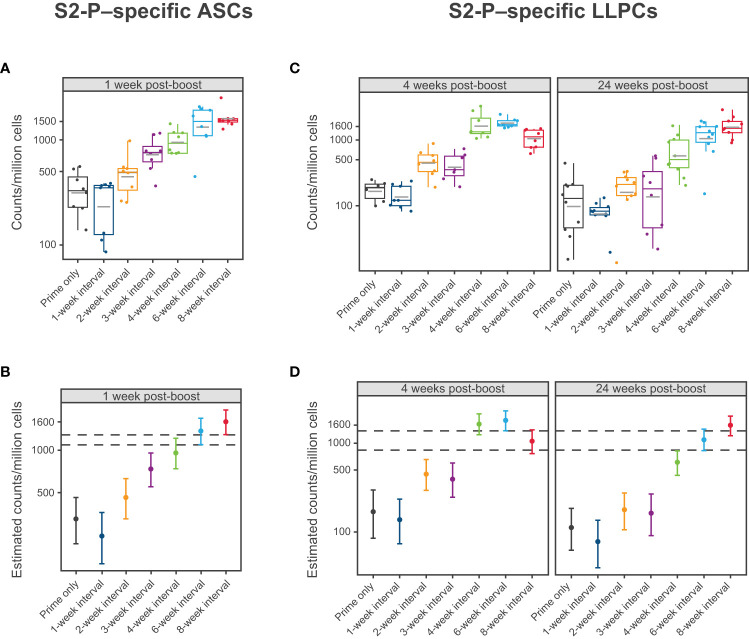
S2-P–specific Antibody Secreting Cells and Long-Lived Plasma Cells. **(A, B)** Levels of S2-P–specific ASCs 1 week following dose 2 of mRNA-1273 10 µg are presented by dosing intervals (prime only or 1-, 2-, 3-, 4-, 6-, or 8-week intervals between doses). Panel **(A)** presents individual animal-level data with dots corresponding to individual animals and grey horizontal lines denoting the average within each group. Panel **(B)** presents the estimated mean and associated 95% CIs based on a statistical model (see Methods), with error bars representing the 95% CI of the estimated mean. **(C, D)** Levels of S2-P–specific LLPCs 4 weeks and 24 weeks after dose 2 of mRNA-1273 10 µg are presented by dosing intervals (prime only or 1-, 2-, 3-, 4-, 6-, or 8-week intervals between doses). Individual animal-level data (dots corresponding to individual animals and grey horizontal line denoting the average within each group) are shown in panel **(C)**. Panel **(D)** presents the corresponding estimated means and associated 95% CIs based on a statistical model (see Methods), with error bars representing the 95% CI of the estimated mean (n=8-10 mice per group). Results of statistical comparisons between groups based on the statistical models are presented in **Table S3**, **Table S4**, and **Figure S6**. ASC, antibody-secreting cell; CI, confidence interval; LLPC, long-lived plasma cell; mRNA, messenger RNA.

To evaluate the impact of the mRNA-1273 dosing interval on S2-P–specific ASC and LLPC induction, mice were immunized with mRNA-1273 10 µg at varying times between dose 1 and 2 and spleens and bone marrow were collected after dose 2 (**Figure 1**). Based on ELISpot assay, 2 doses of mRNA-1273 10 µg administered on 6- and 8-week intervals induced the highest number of S2-P–specific ASCs in spleen 1 week following dose 2 (**Figure 4**; **Table S3**; **Figure S6**). At this time point, the number of S2-P–specific ASCs induced by the 6- and 8-week dosing intervals were generally similar and higher than shorter dosing intervals (≤4 weeks).

At 4 weeks and 24 weeks following dose 2, the 4-, 6-, and 8-week dosing intervals of mRNA-1273 10 µg induced the greatest number of S2-P–specific IgG ASCs (including LLPCs) in the bone marrow (**Figure 4**; **Table S4**; **Figure S6**), although notably, the number of ASCs induced by the 4-week interval declined from 4 weeks to 24 weeks following dose 2, which was not observed with the 6- and 8-week intervals.

### Spike-specific CD4+ and CD8+ T-cell responses

**Figure 5:**
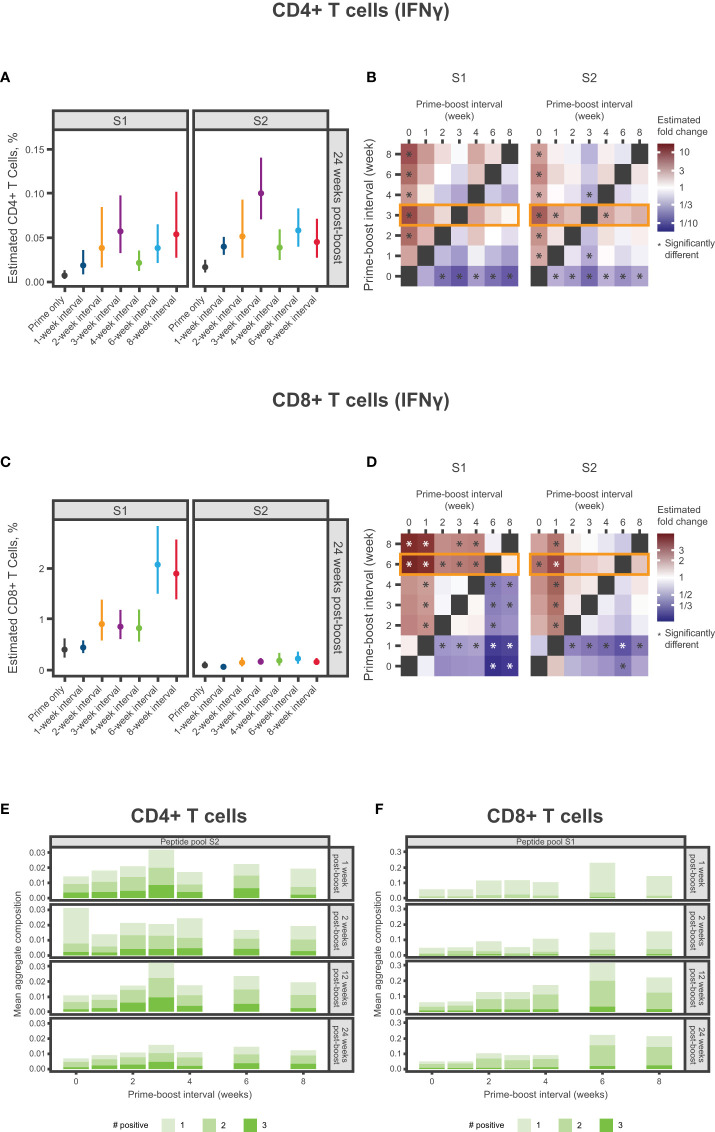
Spike-specific CD4+ and CD8+ T-Cell Responses. **(A-D)** Percentage of SARS-CoV-2 spike-specific IFNγ-producing **(A)** CD4+ T cells or **(C)** CD8+ T cells at 24 weeks after dose 2 according to antigen (S1 and S2) and mRNA-1273 dosing interval (prime only or 1-, 2-, 3-, 4-, 6-, or 8-week intervals). Panels **(A, C)** present the estimated mean and associated 95% CIs based on a statistical model (see Methods), with error bars representing the 95% CIs of estimated means. Panels **(B, C)** present the same results in heatmaps showing fold change between percentages of spike-specific IFNγ-producing **(B)** CD4+ T cells or **(D)** CD8+ T cells at 24 weeks after dose 2. Significant differences denoted by an asterisk if the *P*-value was less than 0.05. Results of statistical comparisons between groups are presented in **Table S5**, **Table S7**, **Figure S7**, and **Figure S10**. **(E, F)** Mean aggregate composition of CD4+ T-cell IFNγ response to the S2 peptide pool or CD8+ T-cell IFNγ response to the S1 peptide pool are presented by mRNA-1273 dosing interval at 1, 2, 12, and 24 weeks following dose 2. Data are presented as averages of individual mice (n=8-10) within each dosing interval group and time point. These compositions were obtained from a thresholding modeling that accounted for day- and cell type–specific differences (see Methods and **Supplementary Methods**). Results of statistical comparisons between groups based on a zero-inflated beta regression model (see Methods) and shown in **Table S6**, **Figure S9**, and **Figure S11**. Individual animal-level data are presented in **Figure S8**. CI, confidence interval; IFNγ, interferon γ; mRNA, messenger RNA; S1, subunit 1; S2, subunit 2; SARS-CoV-2, severe acute respiratory syndrome coronavirus 2.

Mice were immunized with 2 doses of mRNA-1273 10 µg at varying dosing intervals and spike-specific CD4+ and CD8+ T-cell responses were evaluated after dose 2. At 24 weeks after dose 2, the 3-week interval elicited the strongest CD4+ IFNγ response to the S2 peptide pool (**Figure 5**; **Table S5**; **Figure S7**), with a similar trend observed for IL-2 (**Table S5**; **Figure S7**), although responses overall were low. A 3-week interval between first and second doses elicited the highest percentage of polyfunctional CD4+ T helper type 1 (Th1) cells (adjusted *P*-value <0.05; **Figure 5**, **Table S6**; **Figures S8**, **S9**). Both the 6- and 8-week intervals produced the strongest CD8+ IFNγ response to the S1 peptide pool 24 weeks post-dose 2, which were significantly higher than those elicited by a 4-week interval (adjusted *P*-value <0.01; **Figure 5**; **Table S7**; **Figure S10**). A similar trend was also observed for IL-2 and TNF (**Table S7**; **Figure S10**). In CD8+ Th1 cells, the greatest percentage of polyfunctional cells was induced by a 6-week dosing interval of mRNA-1273 (**Figure 5**; **Table S6**; **Figure S11**).

### Anti-PEG antibody levels

**Figure 6:**
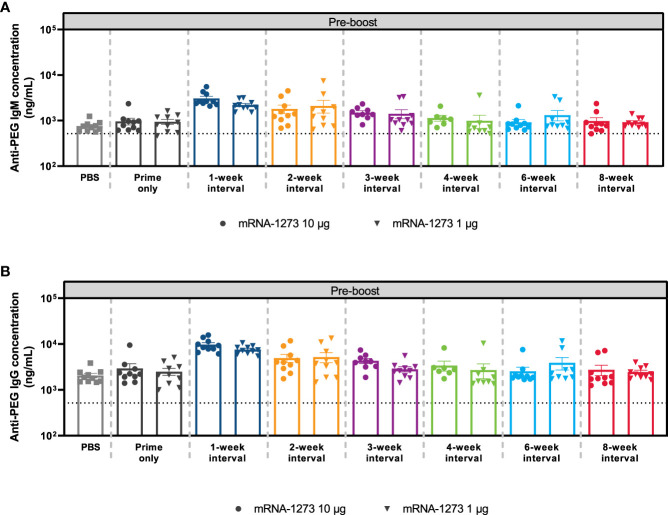
Anti-PEG Antibody Responses. The mean ± SEM concentrations of **(A)** anti-PEG IgM and **(B)** anti-PEG IgG antibodies at Day 56 (before dose 2) are presented by mRNA-1273 dosing interval schedule (prime only or 1-, 2-, 3-, 4-, 6-, and 8-week intervals) with n=8-10 mice per group. Results of corresponding statistical comparisons between groups are shown in **Table S8** and **Figure S12**. IgG, immunoglobulin G; IgM, immunoglobulin M; mRNA, messenger RNA; PEG, polyethylene glycol; PBS, phosphate-buffered saline; SEM, standard error of the mean.

Polyethylene glycol is a hydrophilic, biocompatible polymer that is acknowledged to significantly reduce recognition and clearance of nanoparticles (34, 35). Nevertheless, the generation of anti-PEG antibodies has been associated with considerably faster clearance of PEG-containing drugs and nanocarrier systems upon repeated administration, potentially hindering drug product efficacy (36). Therefore, to evaluate whether elevated levels of anti-PEG antibodies were detected at shorter intervals, mice were immunized with dose 1 of mRNA-1273 (1-µg and 10-µg dose levels) at varying dosing intervals and antibodies to PEG (IgG and IgM) were evaluated before dose 2. Control mice were instead administered PBS. At both the 1-µg and 10-µg dose levels, anti-PEG antibodies (IgG and IgM) were significantly elevated relative to controls after dose 1 and prior to administering dose 2; the greatest anti-PEG antibody titers were observed in mice administered mRNA-1273 1 and 2 weeks (IgG and IgM) and 3 weeks (IgM only) prior to dose 2 (adjusted *P*-value < 0.05; **Figure 6**; **Table S8**; **Figure S12**), which is consistent with the lower levels of immunogenicity observed previously. No significant differences in anti-PEG (IgG and IgM) relative to control were observed for dosing intervals of ≥4 weeks regardless of mRNA-1273 dose level.

## Discussion

This study assessed how the interval between dose 1 and dose 2 of mRNA-1273, an mRNA-based SARS-CoV-2 vaccine, impacted the robustness and durability of immune responses in mice. Overall, longer dosing intervals of mRNA-1273 (ie, 6 to 8 weeks between doses 1 and 2 of the primary vaccination series) generated higher levels of S2-P–specific binding antibodies, higher numbers of S2-P–specific ASCs in the spleen and LLPCs in the bone marrow, as well as increased effector function and polyfunctional CD8+ T cells through 24 weeks following dose 2. Our findings are in agreement with prior studies of mRNA-vaccines as well as other vaccine platforms (15–18, 37, 38), further highlighting that the length of the interval between vaccine prime and boost doses can directly impact multiple aspects of elicited immune responses.

To understand the impact of the dosing interval on immunogenicity elicited by mRNA-1273, we first examined levels of antigen-specific serum binding antibodies through 24 weeks after the second dose. Notably, binding antibody titers elicited by mRNA-1273 have been previously shown as strongly correlated with neutralizing antibody titers (39), a proposed correlate of protection for SARS-CoV-2 vaccines (4, 40). In our study, longer dosing intervals consistently produced more robust S2-P–specific serum binding IgG titers regardless of the mRNA-1273 dose level tested. Relative to levels before dose 2, both 6- and 8-week intervals increased antibody levels at 24 weeks following the second dose, with 30-fold and 10-fold increases for the 1 µg and 10 µg doses, respectively. Overall, the mRNA-1273 10-µg dose level induced greater antibody responses than the 1-µg dose level regardless of the dosing interval; however, this distinction between the 2 different dose levels was reduced over time for animals dosed on a ≥4-week interval, suggesting potential for a dose-sparing effect (**Figure 2B**). To extend upon these findings, we also evaluated ADCC activity, which has been suggested as a correlate of the host immune response to SARS-CoV-2 infection or vaccination (41). Similarly, improved antibody Fc-effector responses were observed with longer (≥4 weeks) over shorter intervals.

The generation of antigen-specific antibodies, produced by ASCs and non-proliferating bone marrow resident LLPCs, is required for durable humoral immunity (42). We therefore evaluated production of S2-P–specific ASCs in the spleen and ASCs including LLPCs in the bone marrow following varying mRNA-1273 dosing intervals. Overall, longer dosing intervals up to 8 weeks elicited higher antigen-reactive ASC counts 1 week after dose 2. Longer dosing intervals were also associated with increased numbers of antigen-reactive ASCs in the bone marrow at 4 weeks and 24 weeks following dose 2, indicative of a more durable long-lived response (42). These results are in alignment with those observed by a clinical study on another mRNA-based SARS-CoV-2 vaccine, BNT162b2, which showed a nearly 7-fold increase in B-cell responses after a 10-week versus a 4-week interval between doses 1 and 2 (17), indicating continued B-cell development beyond 4 weeks after dose 1 and a benefit of longer intervals in the primary dosing regimens.

CD4+ and CD8+ T-cell responses have important roles in resolving SARS-CoV-2 infection and reducing disease severity, with memory T-cells associated with persistent protection over time (43). In this analysis, dosing intervals of 6 weeks or longer elicited the strongest antigen-specific IFNγ CD8+ responses to the S1 peptide pool. Further, measurements of T-cell cytokine polyfunctionality, performed to gain insight to the robustness of the response (44), indicated that the 6-week interval elicited the highest percentage of polyfunctional CD8+ Th1 cells. Comparatively, a dosing interval of 3 weeks elicited the strongest antigen-specific IFNγ CD4+ responses to the S2 peptide pool and the highest percentage of polyfunctional CD4+ Th1 cells, which is consistent with a prior study showing CD4+ T-cell responses to the S2 peptide pool were higher than to the S1 peptide pool in mice vaccinated with 2 doses of mRNA-1273 (45). While it remains difficult to draw overall conclusions, as CD4+ T-cell responses were generally low in our study, it is notable that a recent study in participants older than 80 years found that peak cellular responses after vaccination with the mRNA-based BNT162b2 vaccine against SARS-CoV-2 was observed with the standard 3-week interval as opposed to extended (11-12 weeks) prime-dose intervals (19). However, a separate BNT162b2 real-world study indicated that longer (6- to 14-week) dosing intervals increased IL-2 and IFNγ CD4+ T-cell responses and decreased IFNγ CD8+ T-cell responses (17). Our study also evaluated the impact of the mRNA-1273 dosing interval on immune responses to PEG, a common lipid conjugate of mRNA-LNP vaccines. Anti-PEG antibodies were significantly elevated relative to controls with a dosing interval of 1 and 2 weeks for IgG and 1, 2, and 3 weeks for IgM, which parallels the low levels of immunogenicity detected following dose 2 for these dosing regimens. We speculate that these elevated anti-PEG titers might contribute to the reduced anti-spike antibody responses observed for short dosing intervals. Nevertheless, further investigation is required to evaluate this correlation.

Overall, our findings further illustrate that the interval between vaccine doses can impact mRNA-1273-induced immunogenicity and potentially mRNA vaccines in general. It is notable that during the COVID-19 pandemic, wherein the distribution and administration of safe and efficacious vaccines were paramount to combat a pervasive and deadly disease, it was essential to consider the risk-benefit of the shortest dosing interval of SARS-CoV-2 vaccines that induced potent immune responses and conferred efficacy against infection and disease. However, our findings indicate that a longer (6- to 8-week) interval between mRNA-1273 doses 1 and 2 elicits more robust and durable binding antibody responses with increased effector function and CD8+ T cell polyfunctionality, suggesting that a longer dosing interval could be used to optimize the immune responses elicited by mRNA-1273 in an endemic setting.

Our results are supported by multiple studies reporting that longer dosing intervals for SARS-CoV-2 vaccines may improve vaccine-elicited immunogenicity, effectiveness, and tolerability. A recent study among healthcare workers showed mRNA-1273 induced significantly higher humoral immunogenicity than BNT162b2 regardless of SARS-CoV-2 infection status and age, which the authors suggested might result from the higher antigenic content and longer dosing interval of mRNA-1273 compared with BNT162b2 (4 weeks vs 3 weeks, respectively) (16). Notably, a study found that antibody responses among BNT162b2 recipients were 10-fold higher using a 65- to 84-day versus a 19- to 29-day interval between doses 1 and 2, with consistently higher vaccine effectiveness observed with longer dosing intervals (>45 days) (18). A recent pooled analysis of 4 clinical trials of the ChAdOx1 nCoV-19 adenoviral-vectored vaccine also showed that higher vaccine efficacy against symptomatic COVID-19 was observed, with longer dosing intervals (81.3% at ≥12 weeks vs 55.1% at <6 weeks); antibody responses were also >2-fold higher with a ≥12-week versus <6-week dosing interval (15). Notably, extended intervals between doses 1 and 2 may also limit certain rare safety events observed with currently available COVID-19 mRNA vaccines, with the US Centers for Disease Control and Prevention recently recommending extending the interval between doses 1 and 2 for mRNA-1273 and BNT162b2 (from 4- to 8-week intervals and from 3- to 4-week intervals, respectively) for individuals ≥12 years due to reduced risk of myocarditis with longer intervals (46, 47). Of note, myocarditis and pericarditis following mRNA vaccination are rare and most vaccine-associated myocarditis events have been mild and self-limiting (48, 49).

Limitations to this study include that mouse animal models are not optimal for measuring immune response durability; future studies are planned to further assess the impact of dosing intervals on vaccine-elicited immunogenicity in human participants. Notably, while innate immune responses to SARS-CoV-2 mRNA vaccines differ between mouse animal models and humans, these models are potentially predictive of the innate response and immunogenicity profiles of these vaccines in humans (50). Although this study only examined immune responses elicited by mRNA-1273, it might be anticipated that longer dosing intervals for other mRNA vaccines may similarly elicit more robust and durable immune responses, as has been observed for other vaccine modalities (37, 38).

In conclusion, longer intervals (≥6 weeks) between the first and second vaccine dose of mRNA-1273 induced more durable immune responses in mice. Our findings suggest that extending the current dosing interval could improve immune responses for mRNA-1273 and potentially for other mRNA-based vaccines.

## Data availability statement

The authors declare that the data supporting the findings of this study are available within this article and its Supplementary Information. All R code used to produce the results presented in this article are publicly accessible on the Moderna GitHub repository (https://github.com/modernatx/mRNA1273_dosing_Interval_mice_immune_response_kinetics_quality_magnitude.git).

## Ethics statement

The animal study was reviewed and approved by Institutional Animal Care and Use Committee of Moderna, Inc.

## Author contributions

CH, SE, AC, DE and KB contributed to the study concept and design. Data collection was performed by DG-D, CH, LM, HJ, TM, AF, and HD. All authors besides AC were involved in the analysis and interpretation of the data. All authors were involved in the drafting and critical revision of the manuscript and provided approval for submission. All authors contributed to the article and approved the submitted version.

## Acknowledgments

The authors would like to acknowledge the *In Vivo* Pharmacology team at Moderna, Inc. for assisting in tissue and serum collection. Medical writing and editorial assistance were provided by Emily Stackpole, PhD, and Wynand van Losenoord, MSc, of MEDiSTRAVA in accordance with Good Publication Practice (GPP3) guidelines, funded by Moderna, Inc., and under the direction of the authors.

## Conflict of interest

All authors are employees of Moderna, Inc., and hold stock/stock options from the company. The authors declare that this study received funding from Moderna, Inc. The funder was involved in the study design, collection, analysis, interpretation of data, and the writing of this article or the decision to submit it for publication.

## Publisher's note

All claims expressed in this article are solely those of the authors and do not necessarily represent those of their affiliated organizations, or those of the publisher, the editors and the reviewers. Any product that may be evaluated in this article, or claim that may be made by its manufacturer, is not guaranteed or endorsed by the publisher.

## Abbreviations

ADCC, antibody-dependent cellular toxicity; ASC, antibody secreting cell; COVID-19, coronavirus disease 2019; ELISA, enzyme-linked immunosorbent assay; FBS, fetal bovine serum; GAM, generalized additive model; IFN, interferon; Ig, immunoglobulin; IL, interleukin; LLPC, long lived plasma cell; LNP, lipid nanoparticle; PBS, phosphate-buffered saline; PEG, polyethylene glycol; PerCP, peridinin-chlorophyll-protein; RBD, receptor binding domain; S-2P, SARS-CoV-2 spike protein with 2 proline mutations; SARS-CoV-2, severe acute respiratory syndrome coronavirus 2; Th1, T helper type 1; TMB, 3,3’,5,5’-tetramethylbenzidine; TNF, tumor necrosis factor.
