# Supplementary material for: Altering the mRNA-1273 dosing interval impacts the kinetics, quality, and magnitude of immune responses in mice
Source: Front Immunol. 2022 Nov 8;13:948335. doi: 10.3389/fimmu.2022.948335 (PMC9679967; doi:10.3389/fimmu.2022.948335)
Supplement: Supplementary file 1 [file DataSheet_1.docx]

# *Supplementary Materials*

## 1 Supplementary Methods

### 1.1 Enzyme-linked immunosorbent assay antibody titers time trend estimation

A generalized additive model (GAM) (1) was used to estimate the time trend of antibody titers through 24 weeks following dose 2 for each dosing interval group. A GAM was chosen for its flexibility in capturing both linear and nonlinear trends in the same model. In our analysis, additive terms were used to flexibly estimate the nonlinear trend of post-dose 2 activities in enzyme-linked immunosorbent assay (ELISA) antibodies. We modeled the ELISA data with an equation of the form:

$$\boldsymbol{lo}\boldsymbol{g}_{\boldsymbol{10}}\left( \boldsymbol{titer} \right)_{\boldsymbol{ijk}}\boldsymbol{=}\boldsymbol{\beta}_{\boldsymbol{0}}\boldsymbol{+}\boldsymbol{\beta}_{\boldsymbol{1}}\boldsymbol{interval}_{\boldsymbol{i}}\boldsymbol{+}\boldsymbol{\beta}_{\boldsymbol{2}}\boldsymbol{dose}_{\boldsymbol{k}} {\boldsymbol{+}\boldsymbol{\beta}_{\boldsymbol{3}}\boldsymbol{interval}_{\boldsymbol{i}}\boldsymbol{*day+}\boldsymbol{\beta}_{\boldsymbol{4}}\boldsymbol{interval}_{\boldsymbol{i}}\boldsymbol{*}\boldsymbol{dose}_{\boldsymbol{k}}\boldsymbol{+}\boldsymbol{\beta}_{\boldsymbol{5}}\boldsymbol{dose}_{\boldsymbol{k}}\boldsymbol{*day+}\boldsymbol{\beta}_{\boldsymbol{6}}\boldsymbol{dose}_{\boldsymbol{k}}\boldsymbol{*day*}\boldsymbol{interval}_{\boldsymbol{i}}\boldsymbol{+\beta}}_{\boldsymbol{7}\boldsymbol{i}}\boldsymbol{f}\left( \boldsymbol{day} \right)\boldsymbol{*}\boldsymbol{interval}_{\boldsymbol{i}}\boldsymbol{+}\boldsymbol{\beta}_{\boldsymbol{8}\boldsymbol{k}}\boldsymbol{f}\left( \boldsymbol{day} \right)\boldsymbol{*}\boldsymbol{dose}_{\boldsymbol{k}}\boldsymbol{+}\boldsymbol{\beta}_{\boldsymbol{9}}\boldsymbol{dose}_{\boldsymbol{k}}\boldsymbol{*day+}\boldsymbol{\beta}_{\boldsymbol{10}}\left( \boldsymbol{dose*day*interval} \right)_{\boldsymbol{ij}}\boldsymbol{+}\boldsymbol{\gamma}_{\boldsymbol{0}\boldsymbol{ij}}\boldsymbol{+}{\boldsymbol{\gamma}_{\boldsymbol{1}\boldsymbol{ij}}\boldsymbol{*day+\epsilon}}_{\boldsymbol{ijk}}$$

Where $i,j$ denote dosing interval group, animal $j$ (in group $i$), and dose $k$. Predictors were observed for each animal in each dosing interval group at each dose level. Specifically, the model contained

- Population-level linear trends of categorical and continuous variables
- Two- and three-way interactions of $dose, day$ and $interval$. Including these interactions allows for interval-varying effects of dose levels between days after dose 2.
- A dose-specific smooth nonlinear effect $\boldsymbol{\beta}_{\boldsymbol{8}\boldsymbol{k}}$ in days after dose 2 with a 9-dimensional thin plate spline basis $f\left( day \right);$a dosing interval group-specific smooth nonlinear effect $\boldsymbol{\beta}_{\boldsymbol{7}\boldsymbol{i}}$ in days after dose 2, with a 9-dimensional thin plate spline basis $f\left( day \right).$A maximum degree of freedom of 9 was used based on the unique covariate combinations.
- Animal–specific intercept $\gamma_{0ij}$ and slope $\gamma_{1ij}$

### 1.2 Enzyme-linked immunosorbent assay data fold changes and hypothesis testing

To minimize biases of mean estimation in a nonlinear model, we used analysis of variance and fit days after dose 2 as a categorical variable. Specifically, fold changes of spike-specific serum binding immunoglobulin G (IgG) antibody titers were estimated from the following linear mixed effect model:

$$\begin{aligned} \boldsymbol{lo}\boldsymbol{g}_{\boldsymbol{10}}\left( \boldsymbol{titer} \right)_{\boldsymbol{ijkm}}\boldsymbol{=}\boldsymbol{\beta}_{\boldsymbol{0}}\boldsymbol{+}\boldsymbol{\beta}_{\boldsymbol{1}}\boldsymbol{interval}_{\boldsymbol{i}}\boldsymbol{+}\boldsymbol{\beta}_{\boldsymbol{2}}\boldsymbol{dose}_{\boldsymbol{k}}\boldsymbol{+}\boldsymbol{\beta}_{\boldsymbol{3}}\boldsymbol{day+}\boldsymbol{\beta}_{\boldsymbol{4}}\boldsymbol{interval}_{\boldsymbol{i}}\boldsymbol{*}\boldsymbol{day}_{\boldsymbol{m}}\boldsymbol{+}\boldsymbol{\beta}_{\boldsymbol{5}}\boldsymbol{interval}_{\boldsymbol{i}}\boldsymbol{*}\boldsymbol{dose}_{\boldsymbol{k}} \\ \boldsymbol{+}\boldsymbol{\beta}_{\boldsymbol{6}}\boldsymbol{dose}_{\boldsymbol{k}}\boldsymbol{*}\boldsymbol{day}_{\boldsymbol{m}}\boldsymbol{+}\boldsymbol{\beta}_{\boldsymbol{7}}\boldsymbol{interval}_{\boldsymbol{i}}\boldsymbol{*}\boldsymbol{dose}_{\boldsymbol{k}}\boldsymbol{*}\boldsymbol{day}_{\boldsymbol{m}}\boldsymbol{+}\boldsymbol{\gamma}_{\boldsymbol{0}\boldsymbol{ij}}\boldsymbol{+}\boldsymbol{\epsilon}_{\boldsymbol{ijkm}} \end{aligned}$$

Where $i,j,k$ denote dosing interval group $i$, animal $j$ (in group $i)$and dose level $k$. Predictors were observed for animals j=1, …,8 at $m =$Day 56, Day 64, Day 87, and Day 227 after dose 2. This model estimated $\beta$ for categorical variables *dose*, *interval* and *day*, and animal-specific effect $\gamma_{0ij}$. Two- and three-way interactions for variables $dose, day,$ and $interval$ were included to allow for interval-varying effects. This model was used to accurately estimate means and fold changes while controlling for inter-animal variability.

### 1.3 Antibody-dependent cellular cytotoxicity

A Bayesian GAM was used to capture the time trends in ADCC activity through 24 weeks following dose 2 for each dosing interval group. The observation model took the form:

$$\boldsymbol{log}_{\boldsymbol{2}} \left( \boldsymbol{AUC+1} \right)_{\boldsymbol{ijk}}\boldsymbol{=}\boldsymbol{\beta}_{\boldsymbol{0}}\boldsymbol{+}\boldsymbol{\beta}_{\boldsymbol{1}}\boldsymbol{interva}\boldsymbol{l}_{\boldsymbol{i}}\boldsymbol{+}\boldsymbol{\beta}_{\boldsymbol{2}}\boldsymbol{dos}\boldsymbol{e}_{\boldsymbol{i}}\boldsymbol{+}\boldsymbol{\beta}_{\boldsymbol{3}}\boldsymbol{da}\boldsymbol{y}_{\boldsymbol{i}}\boldsymbol{+}\boldsymbol{\beta}_{\boldsymbol{4}}\left( \boldsymbol{dos}\boldsymbol{e}_{\boldsymbol{i}}\boldsymbol{*interva}\boldsymbol{l}_{\boldsymbol{i}} \right)\boldsymbol{+}\boldsymbol{\beta}_{\boldsymbol{5}}\left( \boldsymbol{dos}\boldsymbol{e}_{\boldsymbol{i}}\boldsymbol{*da}\boldsymbol{y}_{\boldsymbol{i}} \right)\boldsymbol{+}\boldsymbol{\beta}_{\boldsymbol{6}}\left( \boldsymbol{da}\boldsymbol{y}_{\boldsymbol{i}}\boldsymbol{*interva}\boldsymbol{l}_{\boldsymbol{i}} \right)\boldsymbol{+}\boldsymbol{\beta}_{\boldsymbol{7}}\left( \boldsymbol{dos}\boldsymbol{e}_{\boldsymbol{i}}\boldsymbol{*da}\boldsymbol{y}_{\boldsymbol{i}}\boldsymbol{*interva}\boldsymbol{l}_{\boldsymbol{i}} \right)\boldsymbol{+}{\boldsymbol{\beta}_{\boldsymbol{8}\boldsymbol{i}}\boldsymbol{f}\left( \boldsymbol{day} \right)\boldsymbol{+}\boldsymbol{\beta}_{\boldsymbol{9}\boldsymbol{l}}\boldsymbol{h}\left( \boldsymbol{dose} \right)\boldsymbol{+}\boldsymbol{\epsilon}_{\boldsymbol{jk}}}$$

Where *I*, *j*, *k*, and *l* index dosing interval group, sample, and day after dose 2 (in group *i*), and dose for the ADCC data. We allowed the residual variance $\epsilon_{ijk}$ to change as a linear function of *k*. Dosing interval- and dose-specific smooth nonlinear trends in days after dose 2 were captured via 9-dimensional thin plate spline bases *f*(*day*) and *h*(*dose*). Dose and dosing interval were treated here as factors, while day was treated as a continuous variable in order to estimate the smooth trends.

### 1.4 Anti-polyethylene glycol antibody levels

Anti-polyethylene glycol (PEG) antibody levels were analyzed using linear mixed effects models, separately for immunoglobulin M (IgM) and IgG. Specifically, for each antibody, we fit the model:

$${\mathbf{log}_{\boldsymbol{2}} \left( \boldsymbol{tite}\boldsymbol{r}_{\boldsymbol{ijk}} \right)\boldsymbol{=}\boldsymbol{\beta}_{\boldsymbol{0}}\boldsymbol{+}\boldsymbol{\beta}_{\boldsymbol{1}}\boldsymbol{grou}\boldsymbol{p}_{\boldsymbol{i}}\boldsymbol{+}\boldsymbol{\gamma}_{\boldsymbol{j}}\boldsymbol{+\epsilon}}_{\boldsymbol{ijk}}$$

Where *i,j,k* index dosing interval group, dose level, and individual animal; *group* refers to the dosing interval group and $\gamma_{j}$ denotes the random intercept for dose level *j*.

### 1.5 S2-P spike-specific antibody-secreting cell and long-lived plasma cell analysis

Generalized linear regression was used for spike-specific antibody-secreting cell (ASC) and long-lived plasma cell (LLPC) analysis. We transformed ASC counts/million cells using a Box-Cox transformation (2) and modeled the transformed count by $g(counts)= \beta_{0}+\beta_{1}interval$ for both spleen and bone marrow data, where $g(.)$ denotes Box-Cox transformation.

### 1.6 Cytokine polyfunctionality data­­

First, we used SPICE to normalize individual animal measurements (3). Next, we determined the signal threshold by fitting a mixture model. Specifically, the SPICE-normalized composition data contains a background signal that is normally distributed at about 0. The goal of thresholding is to set small positive and negative values to 0. We applied thresholding to compositions *x* in a cell type-, peptide-, and day (after dose 2)-specific manner using a statistical model:

$$\boldsymbol{x}_{\boldsymbol{ijkl}}\boldsymbol{\sim}\boldsymbol{\pi}_{\boldsymbol{ijk}}^{\boldsymbol{0}}\boldsymbol{\cdot Normal}\left( \boldsymbol{0,}\boldsymbol{\sigma}_{\boldsymbol{ijk}}^{\boldsymbol{2}} \right)\boldsymbol{+}\left( \boldsymbol{1-}\boldsymbol{\pi}_{\boldsymbol{ijk}}^{\boldsymbol{0}} \right)\boldsymbol{\cdot Beta}\left( \boldsymbol{\alpha}_{\boldsymbol{ijk}}\boldsymbol{,}\boldsymbol{\beta}_{\boldsymbol{ijk}} \right)$$

Where *i*, *j*, *k*, and *l* index cell type, peptide, day after dose 2, and animal, respectively, and $\pi_{ijk}^{0}\in[0, 1]$. This model assumed that $\pi_{ijk}^{0}$ is the proportion of signal that is background, that the background signal is normally distributed about 0, and that the true non-zero signals are beta-distributed. The threshold within each cell type, peptide, and day was set to be the 95^th^ quantile of the normal component (ie, the background component) of the mixture. Parameters $\pi_{ijk}^{0}$, $\sigma_{ijk}^{2}$, $\alpha_{ijk}$, and $\beta_{ijk}$ were estimated using the expectation-maximization algorithm (4). $L^{signal}(x; \theta^{\left( t \right)})$ and $L^{background}(x; \theta^{\left( t \right)})$ were the likelihood function of data *x* in the signal and background models, respectively, given current parameter estimates at iteration *t*, $\theta^{(t)}$. Then, in the E-step, we calculated:

$\boldsymbol{P}\left( \boldsymbol{Z}_{\boldsymbol{ijkl}}\boldsymbol{=background} \mid\boldsymbol{X}_{\boldsymbol{ijk}}\boldsymbol{=}\boldsymbol{x}_{\boldsymbol{ijk}}\boldsymbol{;}\boldsymbol{\theta}^{\boldsymbol{(t)}} \right)\boldsymbol{=}\frac{\boldsymbol{\pi}_{\boldsymbol{ijk}}^{\boldsymbol{0(t)}}\boldsymbol{L}^{\boldsymbol{background}}\boldsymbol{(}\boldsymbol{x}_{\boldsymbol{ijk}}\boldsymbol{;}{\boldsymbol{\sigma}_{\boldsymbol{ijk}}^{\boldsymbol{2}}\boldsymbol{= \sigma}}_{\boldsymbol{ijk}}^{\boldsymbol{2}\left( \boldsymbol{t} \right)}\boldsymbol{)}}{\boldsymbol{\pi}_{\boldsymbol{ijk}}^{\boldsymbol{0(t)}}\boldsymbol{L}^{\boldsymbol{background}}\left( \boldsymbol{x}_{\boldsymbol{ijk}}\boldsymbol{;}{\boldsymbol{\sigma}_{\boldsymbol{ijk}}^{\boldsymbol{2}}\boldsymbol{= \sigma}}_{\boldsymbol{ijk}}^{\boldsymbol{2}\left( \boldsymbol{t} \right)} \right)\boldsymbol{+}\left( \boldsymbol{1-}\boldsymbol{\pi}_{\boldsymbol{ijk}}^{\boldsymbol{0}\left( \boldsymbol{t} \right)} \right)\boldsymbol{L}^{\boldsymbol{signal}}\boldsymbol{(}\boldsymbol{x}_{\boldsymbol{ijk}}\boldsymbol{;}\boldsymbol{\alpha}_{\boldsymbol{ijk}}\boldsymbol{=}\boldsymbol{\alpha}_{\boldsymbol{ijk}}^{\left( \boldsymbol{t} \right)}\boldsymbol{,}\boldsymbol{\beta}_{\boldsymbol{ijk}}\boldsymbol{=}\boldsymbol{\beta}_{\boldsymbol{ijk}}^{\boldsymbol{(t)}}\boldsymbol{)}}$

$$\boldsymbol{P}\left( \boldsymbol{Z}_{\boldsymbol{ijkl}}\boldsymbol{=signal} \mid\boldsymbol{x}_{\boldsymbol{ijk}}\boldsymbol{=}\boldsymbol{X}_{\boldsymbol{ijk}}\boldsymbol{;}\boldsymbol{\theta}^{\boldsymbol{(t)}} \right)\boldsymbol{=1-P}\left( \boldsymbol{Z}_{\boldsymbol{ijkl}}\boldsymbol{=background} \mid{\boldsymbol{x}_{\boldsymbol{ijk}}\boldsymbol{= X}}_{\boldsymbol{ijk}}\boldsymbol{;}\boldsymbol{\theta}^{\boldsymbol{(t)}} \right)$$

Where $\boldsymbol{Z}_{\boldsymbol{ijkl}}$ is the unknown label (signal or background) of the *l*^th^ animal for cell type *i*, peptide *j*, at the *k*^th^ day after dose 2 and $\boldsymbol{X}_{\boldsymbol{ijk}}$ is the data. In the M-step, we updated:

$${\boldsymbol{\pi}_{\boldsymbol{ijk}}^{\boldsymbol{0}}}^{\boldsymbol{(t+1)}}\boldsymbol{=1/n}\sum_{\boldsymbol{l=1}}^{\boldsymbol{n}} \boldsymbol{P(}\boldsymbol{Z}_{\boldsymbol{ijkl}}\boldsymbol{=background\mid}\boldsymbol{x}_{\boldsymbol{ijk}}\boldsymbol{=}\boldsymbol{X}_{\boldsymbol{ijk}}\boldsymbol{)}$$

$${\boldsymbol{\sigma}_{\boldsymbol{ijk}}}^{\boldsymbol{(t+1)}}\boldsymbol{=}\sqrt{\boldsymbol{[}\sum_{\boldsymbol{l}} \boldsymbol{x}_{\boldsymbol{ijkl}}^{\boldsymbol{2}}\boldsymbol{P(}\boldsymbol{Z}_{\boldsymbol{ijkl}}\boldsymbol{=background\mid}\boldsymbol{x}_{\boldsymbol{ijk}}\boldsymbol{=}\boldsymbol{X}_{\boldsymbol{ijk}}\boldsymbol{)]/[}\sum_{\boldsymbol{l}} \boldsymbol{P(}\boldsymbol{Z}_{\boldsymbol{ijkl}}\boldsymbol{=background\mid}\boldsymbol{x}_{\boldsymbol{ijk}}\boldsymbol{=}\boldsymbol{X}_{\boldsymbol{ijk}}\boldsymbol{)}\boldsymbol{]}}$$

The beta distribution parameters $\alpha_{ijk}$ and $\beta_{ijk}$ were updated numerically to maximize the function:

$\sum_{\boldsymbol{l=1}}^{\boldsymbol{n}} \left( \boldsymbol{1-}\boldsymbol{\pi}_{\boldsymbol{ijk}}^{\boldsymbol{0}} \right)\boldsymbol{L}^{\boldsymbol{signal}}\left( \boldsymbol{x}_{\boldsymbol{ijkl}}\boldsymbol{;}\boldsymbol{\alpha}_{\boldsymbol{ijk}}^{\left( \boldsymbol{t} \right)}\boldsymbol{,}\boldsymbol{\beta}_{\boldsymbol{ijk}}^{\boldsymbol{(t)}} \right)\boldsymbol{\times}\boldsymbol{\pi}_{\boldsymbol{ijk}}^{\boldsymbol{0}}\boldsymbol{L}^{\boldsymbol{background}}\boldsymbol{(}\boldsymbol{x}_{\boldsymbol{ijkl}}\boldsymbol{;}\boldsymbol{\sigma}_{\boldsymbol{ijk}}^{\boldsymbol{2(t)}}\boldsymbol{)}$.

The thresholded data were analyzed using a zero-inflated beta regression model (5). When *y* = 0, this model took the form:

***f(y)=ν***

And when *y >* 0, the model took the form:

***f*(*y*∣*μ*,*σ*)=(1−*ν*)[Γ(*μσ*)Γ(*σ*)]/[Γ((1−*μ*)⋅*σ*)]​*y*^(^*^μσ^*^−1)^(1−*y*)^(((1−^*^μ^*^)^*^σ^*^)−1)^**

Where 0 < *μ* < 1, *σ* > 0, 0 < *ν* <1, and *y* is a thresholded composition.

### 1.7 Spike-specific CD4+ and CD8+ T cell IFNγ responses

Zero-inflated beta regression was similarly used to model spike-specific CD4+ and CD8+ T-cell IFNγ responses. We modeled the mean component as spike-specific CD 4+ and CD8+ T-cell responses with day and interval groups as explanatory variables. We included the interaction between day and dosing interval group when it was estimable. To account for heterogeneity, we modeled the scale parameter using the same explanatory variables as with the mean component.

## 2. Supplementary Figures

### Supplementary Figure 1. Impact of mRNA-1273 Prime-boost Interval on S2-P-specific Serum Binding IgG Antibody Titers Through 24 Weeks After Dose 2.

(A) S2-P serum binding IgG antibody titers from before dose 2 through 24 weeks following dose 2 are presented according to mRNA-1273 dosing level (1 µg or 10 µg) and prime-boost interval group (prime only or 1-, 2-, 3-, 4-, 6-, or 8-week intervals between doses). Individual animal-level data are shown (n=8-10 mice per group) with dots corresponding to individual animals and boxplots indicating quartiles of log10 S2-P IgG titers within each group. Only non-terminal groups are shown. The dotted line represents the LLOQ. (B) S2-P serum binding IgG antibody titers from before dose 2 through 24 weeks following dose 2 are presented according to mRNA-1273 dosing level (1 µg or 10 µg) and prime-boost interval group (prime only or 1-, 2-, 3-, 4-, 6-, or 8-week intervals between doses). Individual animal-level data are shown (n=8-10 mice per group).

IgG, immunoglobulin G; LLOQ, lower limit of quantitation; mRNA, messenger RNA; wk, week.


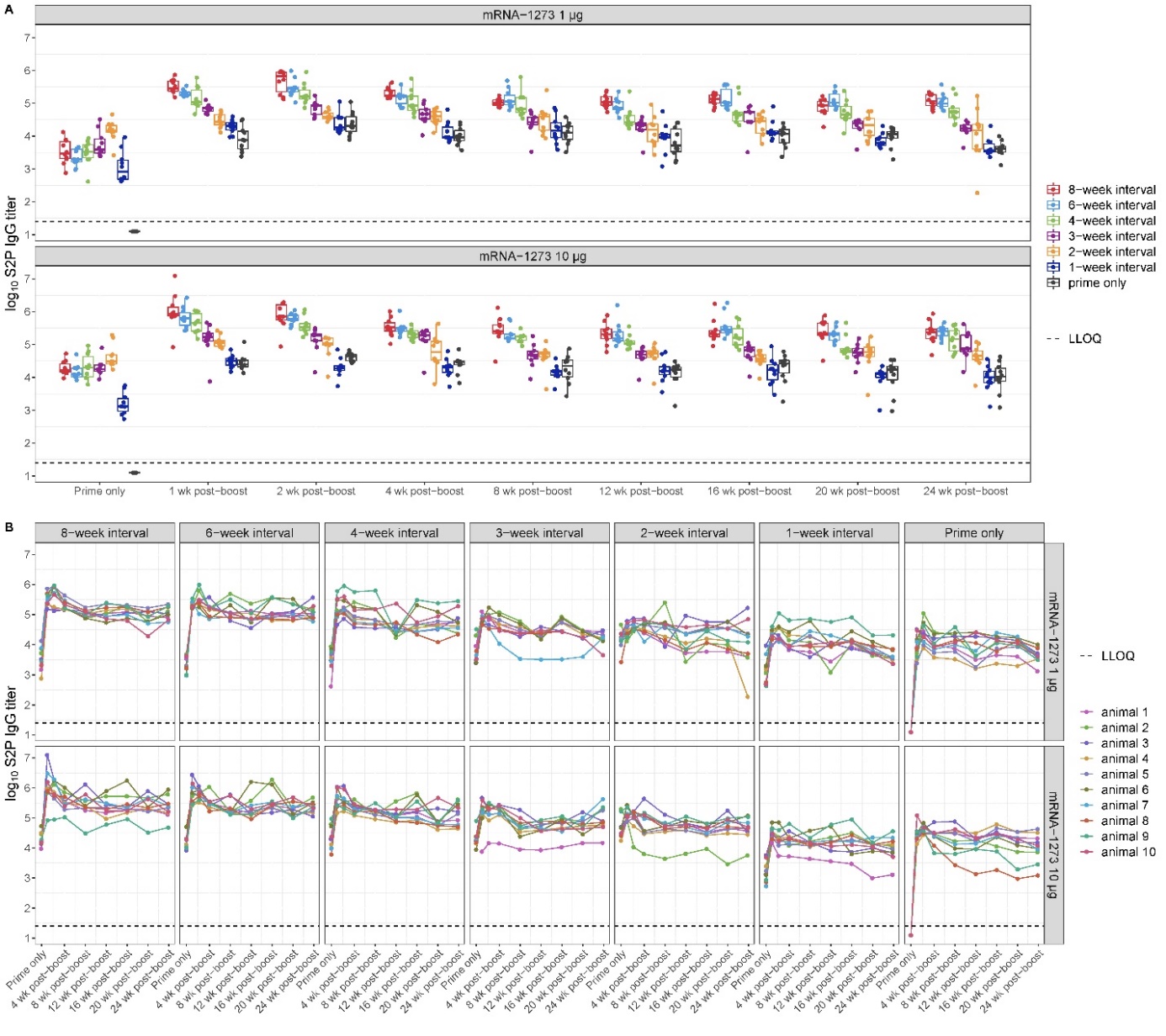


### Supplementary Figure 2. Predicted Fold Change of S2-P-specific Serum Binding IgG Antibody Titers.

The fold change of S-2P-specific serum-binding IgG antibody titers from 2 weeks after versus before dose 2 or from 24 weeks after versus before dose 2 are presented by mRNA-1273 dosing interval (1-, 2-, 3-, 4-, 6-, or 8-week intervals) and dose level (1 µg or 10 µg). Data shown indicate estimated means based on a statistical model, with error bars reflecting 95% CI of the estimated means. The dotted line indicates fold-change of 1, which corresponds to no difference between groups compared.

CI, confidence interval; IgG, immunoglobulin G; mRNA, messenger RNA.


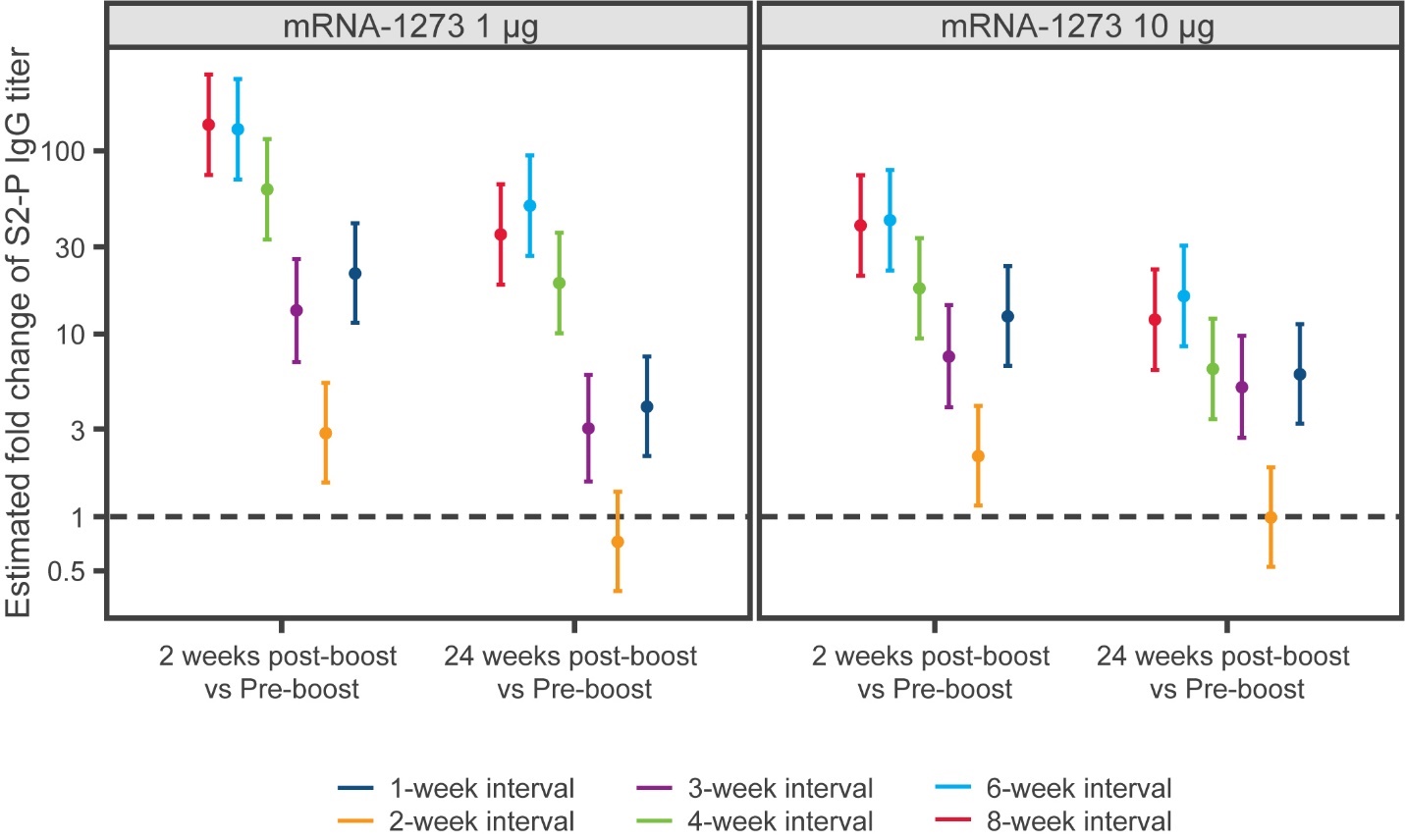


### Supplementary Figure 3. Statistical Comparisons of S2-P-specific Serum Binding IgG Antibody Titers Between Different mRNA-1273 Dosing Intervals.

Comparison X/Y refers to the fold change in S2-P-specific serum binding IgG antibody titers of the X-week dosing interval group compared with the Y-week dosing interval group. Dots represent the estimated fold changes based on the GAM, and error bars representing their associated 95% CI. Comparisons are considered statistically significant when 95% CIs do not cover 1. The dotted vertical line indicates fold-change of 1, which corresponds to no difference between groups compared. This figure is a visual analog of the *P*-values reported in **Table S1**.

CI, confidence interval; IgG. immunoglobulin G; mRNA, messenger RNA; wk, week

### Supplementary Figure 4. Animal-level Antibody Fc-effector Function Responses.

As an expansion of **Figure 3**, individual animal-level antibody Fc-effector function responses are shown for each mRNA-1273 dosing interval and dose level (1 µg or 10 µg) from before dose 2 to 24 weeks after dose 2. Horizontal gray dashes indicate the geometric mean for the AUC within each group and post-boost timepoint.

AUC, area under the curve; mRNA, messenger RNA; wk, week.

**
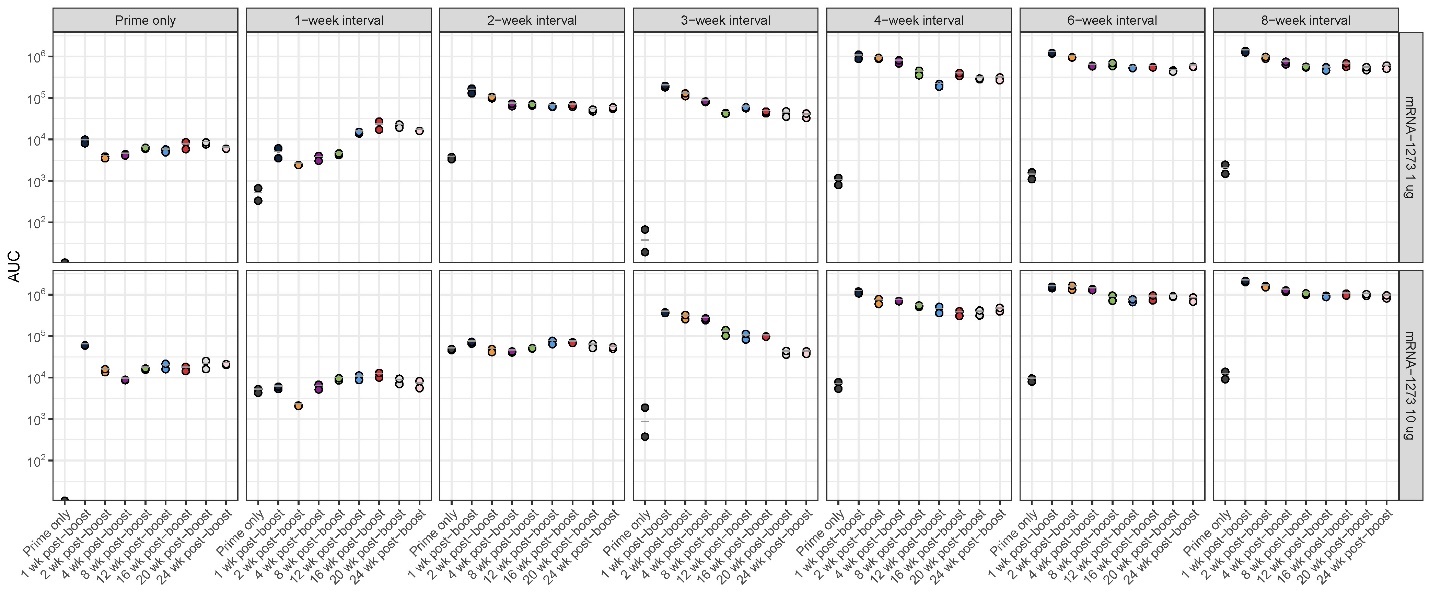
**

### Supplementary Figure 5. Statistical Comparisons of Antibody Fc-effector Function Responses Between mRNA-1273 Dosing Intervals.

Comparisons X/Y refer to the fold change in AUC of the X-week dosing interval group compared to the Y-week dosing interval group. Comparisons were considered statistically significant when the lower limit of 95% CIs was >1 (one-sided 95% CIs). The dotted vertical red line indicates fold-change of 1, which corresponds to no difference between groups compared. This figure is a visual analog of the *P*-values presented in **Table S2**.

AUC, area under the curve; CI, confidence interval; mRNA, messenger RNA.

******

### Supplementary Figure 6. Statistical Comparisons of S2-P-specific Antibody Secreting Cells and Long-Lived Plasma Cells Between 10 µg mRNA-1273 Dosing Intervals

(A) Statistical comparisons of S2-P-specific ASCs at 1 week after dose 2 between mRNA-1273 10 µg dosing intervals. (B) Statistical comparisons of S2-P-specific LLPCs between mRNA-1273 10 µg dosing intervals at 4 and 24 weeks after dose 2. Dots in each panel are representative of the estimated fold change based on a statistical model, with error bars representing the 95% CIs. The dotted vertical green line indicates fold-change of 1, which corresponds to no difference between groups compared. This figure is a visual analog of the *P*-values reported in **Table S3** and **Table S4**.

CI, confidence interval; mRNA, messenger RNA; wk, week.


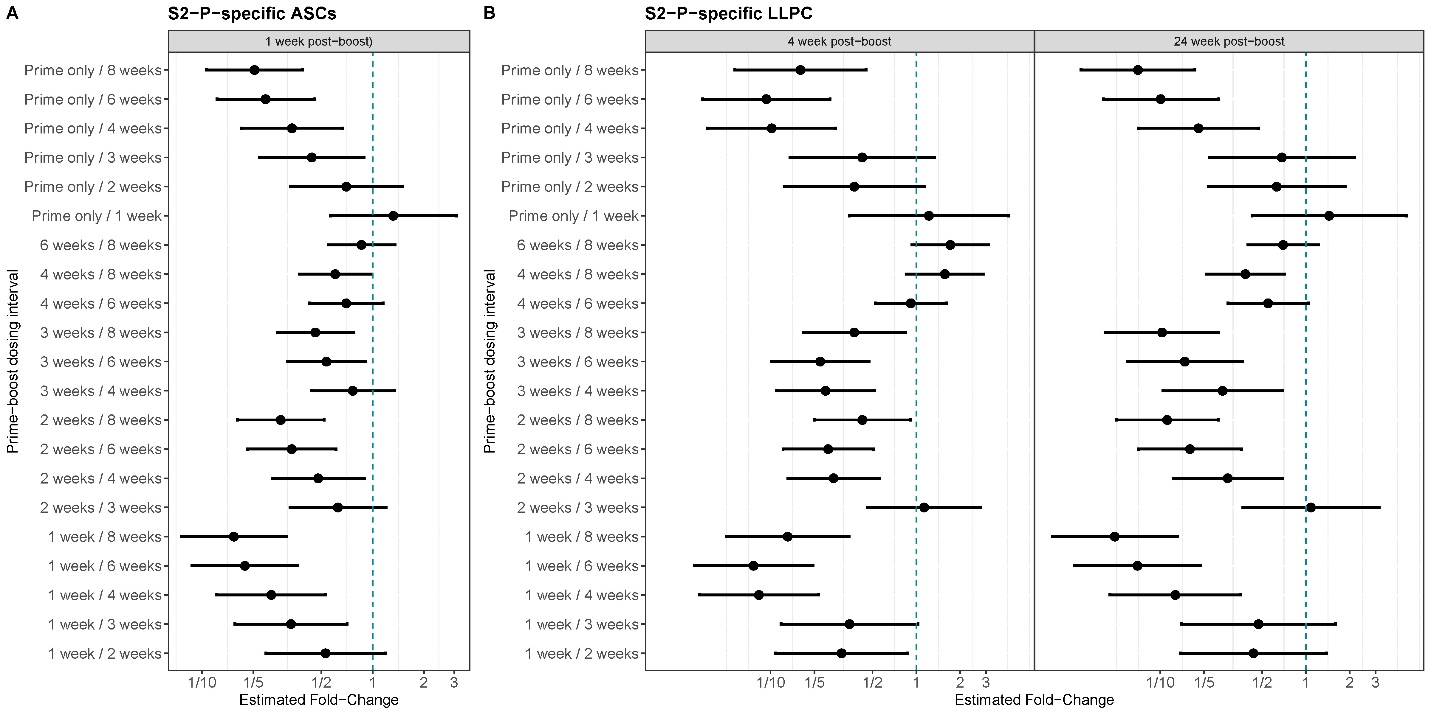


### Supplementary Figure 7. Statistical Comparisons of the Percentage of SARS-CoV-2 Spike-specific IFNγ, IL-2, and TNFα Producing CD4+ T Cells Through 24 Weeks After Dose 2.

Comparison X/Y refers to the fold change in the percentage of SARS-CoV-2 spike-specific INFγ, IL-2, and TNFα producing CD4+ T cells in the X-week dosing interval group over the Y-week dosing interval group. Dots are representative of the estimated fold change based on a statistical model, with error bars representing the 95% CIs. Comparisons are considered statistically significant when 95% CIs >1. This figure is a visual analog of the *P*-values reported in **Table S5**.

CI, confidence interval; IFNγ, interferon γ; IL-2, interleuklin-2; mRNA, messenger RNA; S1, subunit 1; S2, subunit 2; SARS-CoV-2, severe acute respiratory syndrome coronavirus 2; TNFα, tumor necrosis factor α.


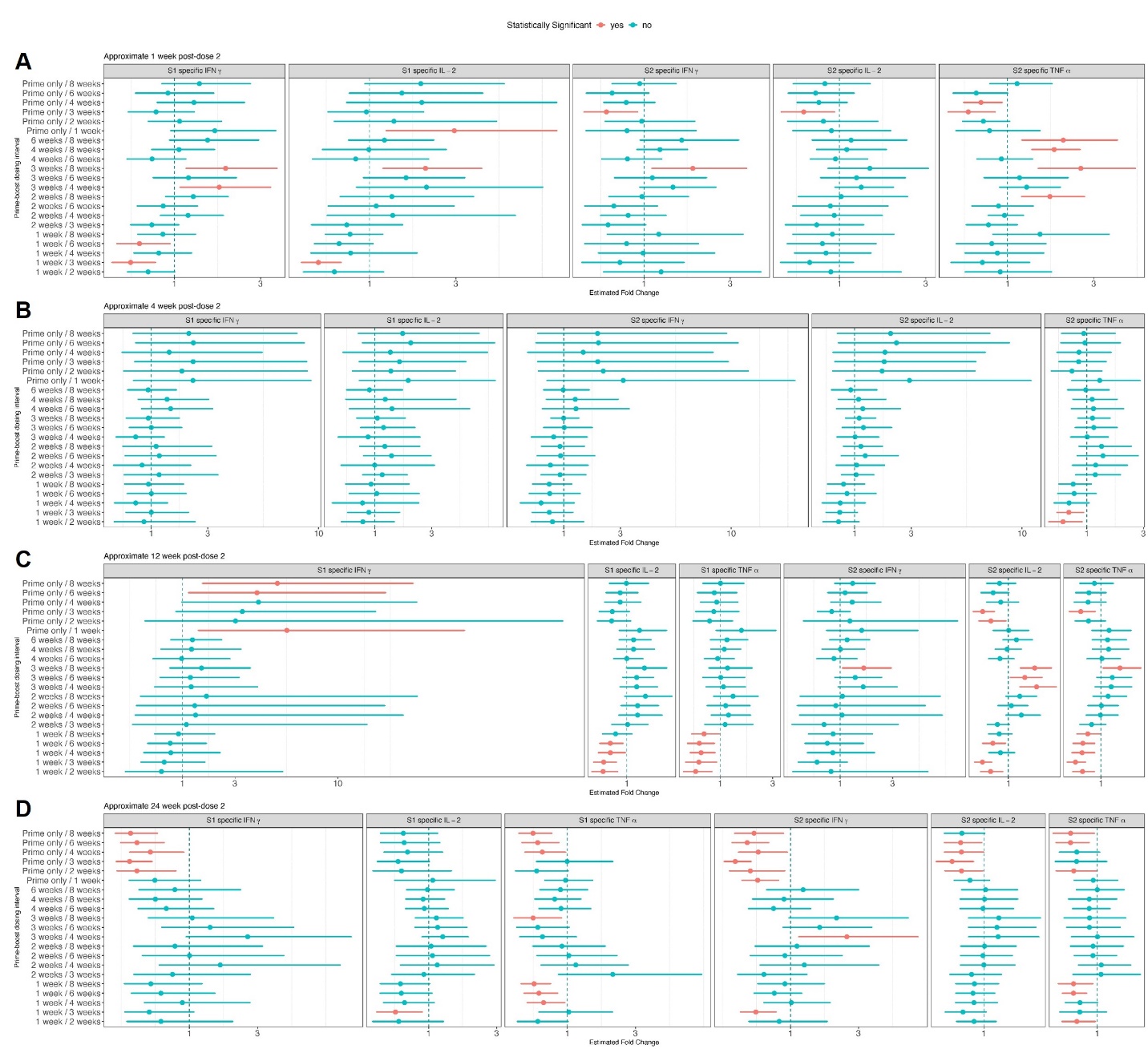


### Supplementary Figure 8. Animal-level Spike-specific CD4+ and CD8+ T-Cell Responses.

Thresholded aggregate composition of individual level (n=8-10 mice per group) CD4+ T-cell polyfunctional cytokine response to the S2 peptide pool and CD8+ T-cell polyfunctional cytokine response to the S1 peptide pool are presented by mRNA-1273 dosing interval schedule (prime only or 1-, 2-, 3-, 4-, 6-, or 8-week intervals between doses) at 1-, 2-, 12-, and 24-weeks following dose 2. Data are presented with y-axis on square root scale for ease of viewing. Thresholding of normalized compositions was performed using a flexible statistical model that also accounted for day- and cell type-specific differences (see **Supplementary Methods**).

mRNA, messenger RNA; S1, subunit 1; S2, subunit 2.


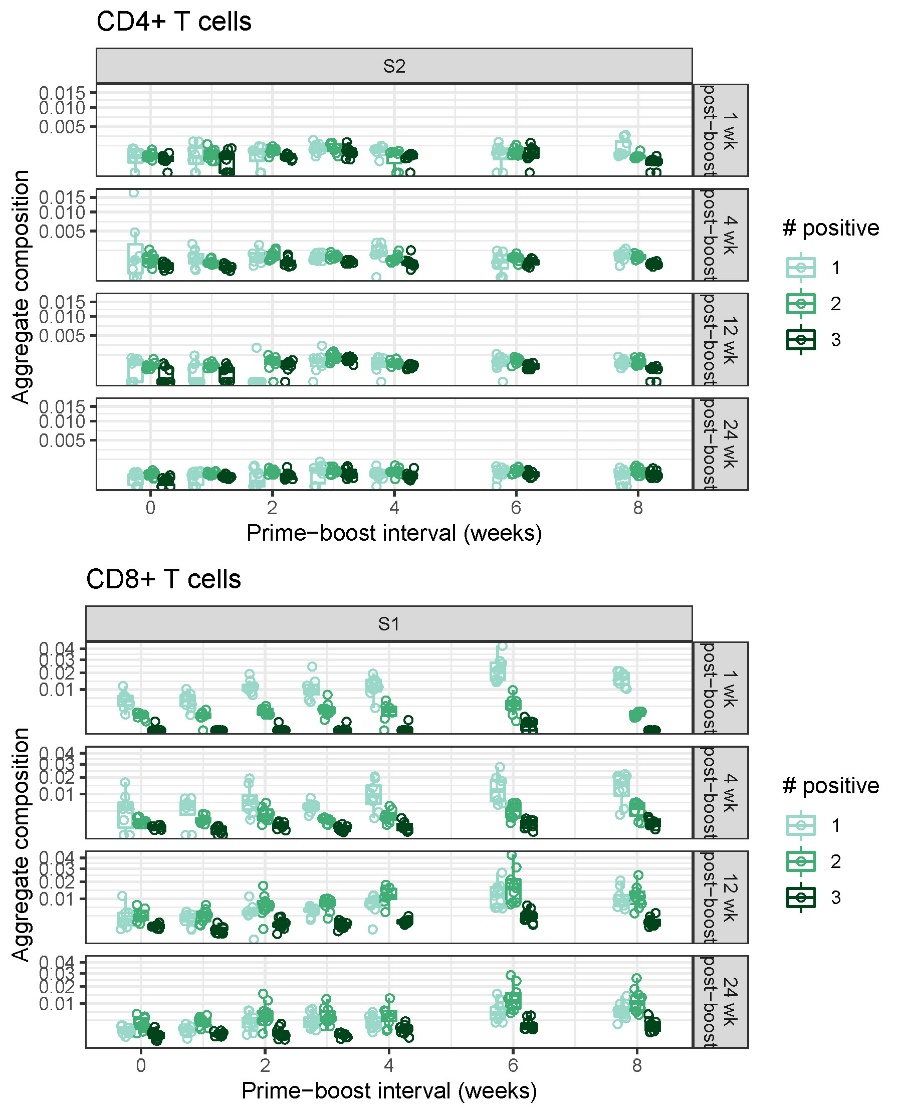


### Supplementary Figure 9. Statistical Comparisons of CD4+ T Cell Cytokine Polyfunctionality.

Statistical comparisons of single, dual, and triple cytokine expressing CD4+ T cells by dosing interval and time after dose 2. Comparison X/Y assesses the odds ratio of group X expressing 1, 2, or 3 cytokines over group Y. Dots represent estimated odds ratio based on the statistical model, with error bars representing the 95% CIs. Statistical comparisons are statistically significant when 95% CIs do not cover 1. Group X is significantly more (less) likely to express 1, 2, or 3 cytokines when the CI lies entirely above (below) 1. This figure is a visual analog of the *P*-values presented in **Table S6**.

CI, confidence interval.


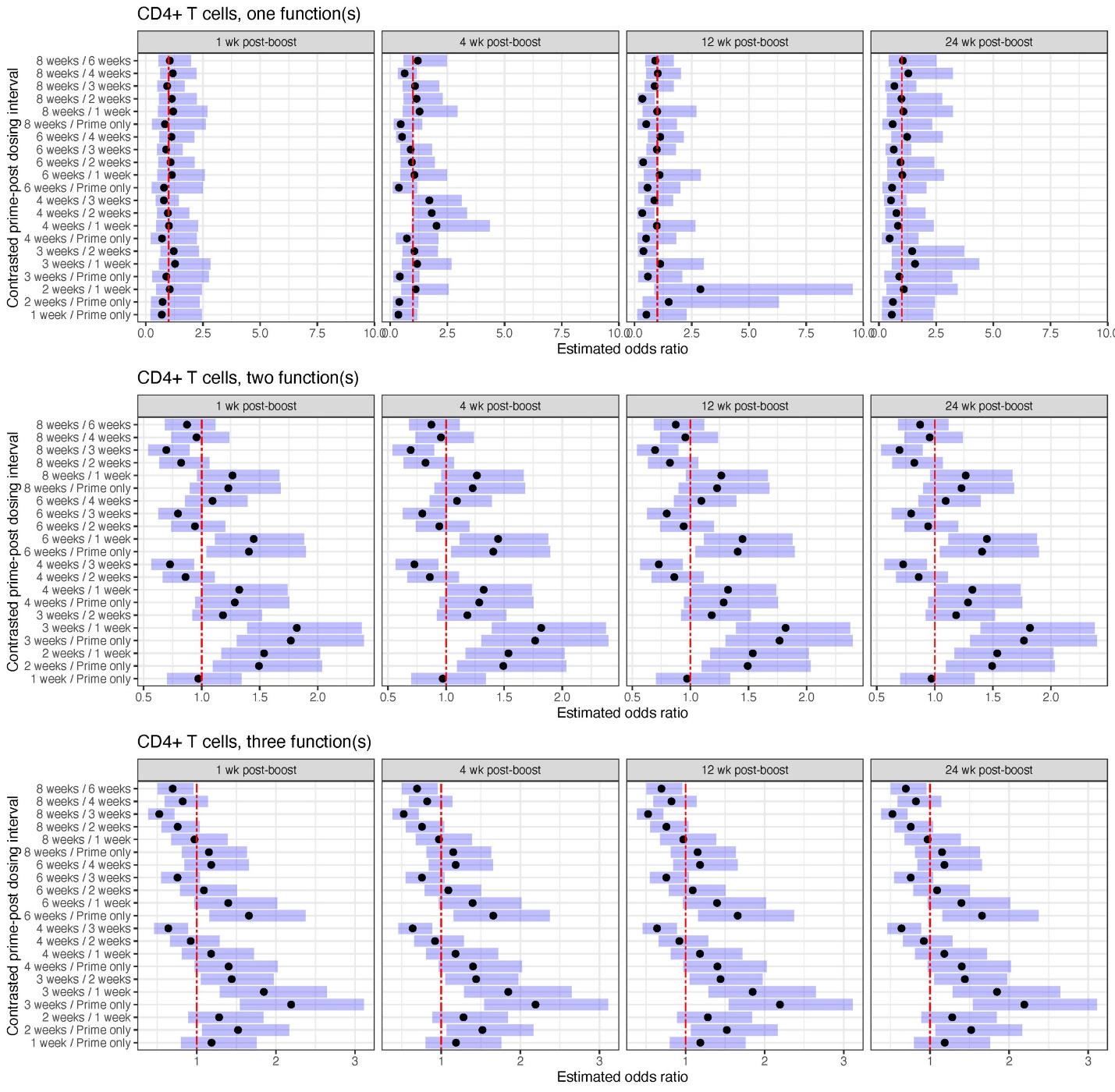


### Supplementary Figure 10. Statistical Comparisons of Percentage of SARS-CoV-2 Spike-specific IFNγ, IL-2, and TNFα Producing CD8+ T Cells Through 24 Weeks After Dose 2.

Comparison X/Y refers to the fold change in percentage of SARS-CoV-2 spike-specific INFγ, IL-2, and TNFα producing CD8+ T cells in the X-week dosing interval over the Y-week dosing interval group. Dots are representative of the estimated fold change based on a statistical model, with error bars reflecting the 95% CIs. Statistical comparisons are statistically significant when 95% CIs do not cover 1. Significant comparisons are shown in red. This figure is a visual analog to *P*-values reported in **Table S7**.

CI, confidence interval; IFNγ, interferon γ; IL-2, interleuklin-2; mRNA, messenger RNA; S1, subunit 1; S2, subunit 2; SARS-CoV-2, severe acute respiratory syndrome coronavirus 2; TNFα, tumor necrosis factor α.


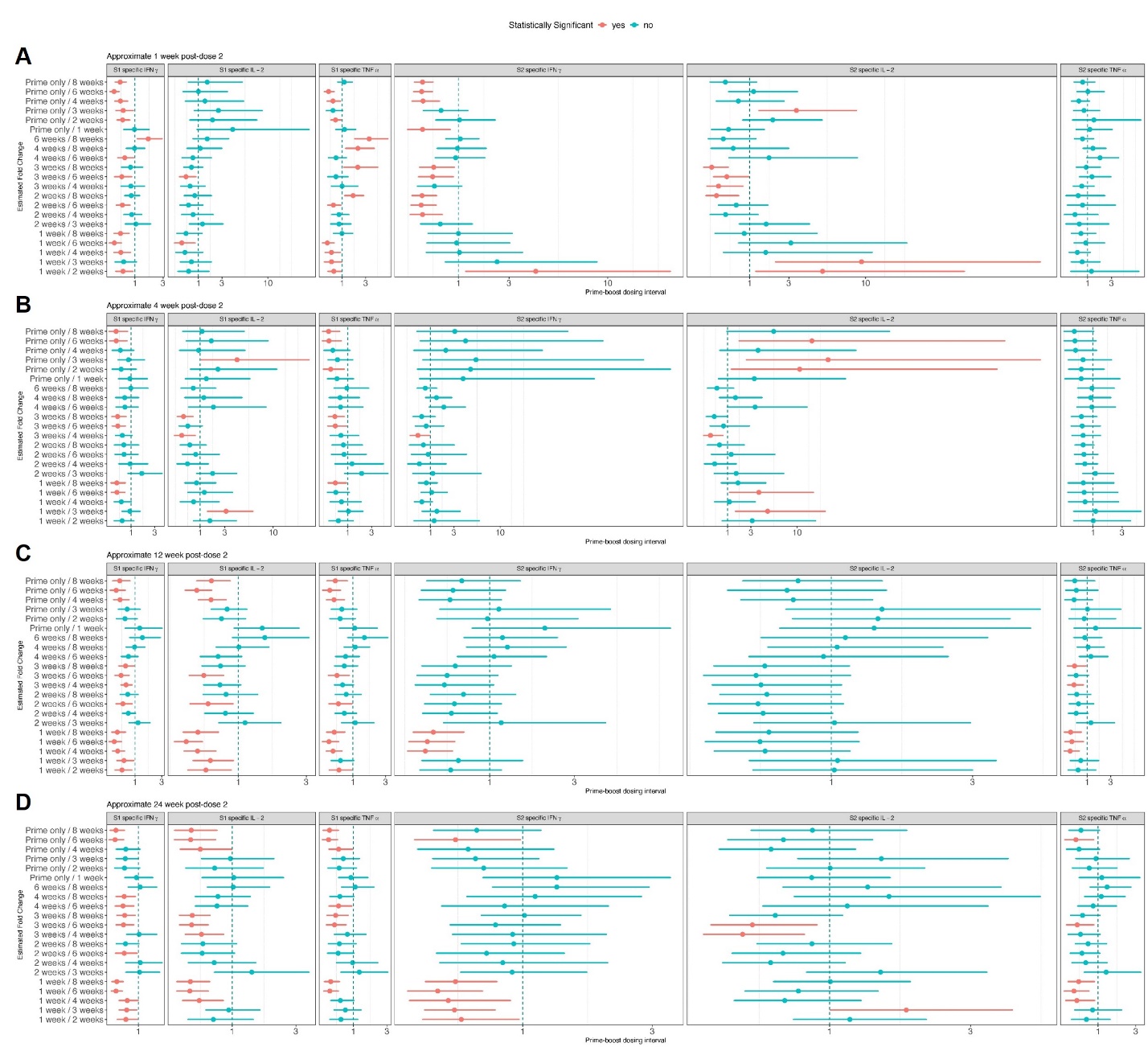


### Supplementary Figure 11. Statistical Comparisons of CD8+ T Cell Cytokine Polyfunctionality.

Statistical comparisons of single, dual, and triple cytokine expressing CD8+ T cells by dosing interval and time post boost. Comparison X/Y assesses the odds ratio of group X expressing 1, 2, or 3 cytokines over group Y. Dots are representative of the estimated fold change based on a statistical model, with error bars reflecting the 95% CIs. Statistical comparisons are statistically significant when 95% CIs do not cover 1. Group X is significantly more (less) likely to express 1, 2, or 3 cytokines when the CI lies entirely above (below) 1. This figure is a visual analog of the *P*-values presented in **Table S6**.

CI, confidence interval.


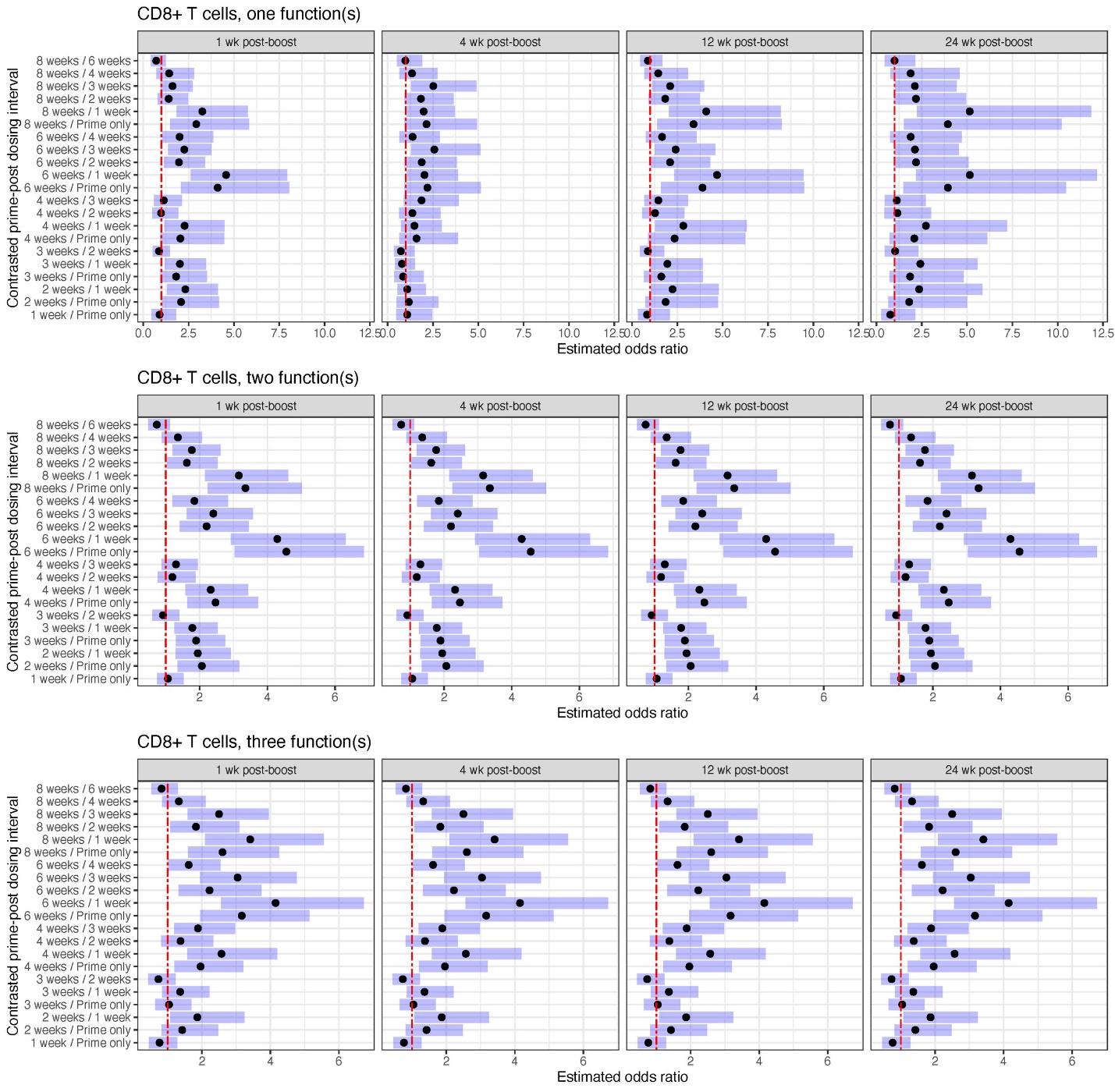


### Supplementary Figure 12. Statistical Comparisons of Anti-PEG IgG and IgM Antibody Responses Between mRNA-1273 Dosing Intervals Over Control.

Comparison X/Y assesses the fold change in anti-PEG antibody concentrations of the X-week dosing interval group over the PBS control. Dots are representative of the estimated fold change based on a statistical model, with error bars reflecting the 95% CIs. Statistical comparisons are statistically significant when 95% CIs do not cover 1. This figure is a visual analog of the *P*-values reported in **Table S8**.

CI, confidence interval; IgG, immunoglobulin G; IgM, immunoglobulin M; mRNA, messenger RNA; PBS, phosphate-buffered saline; PEG, polyethylene glycol.

***
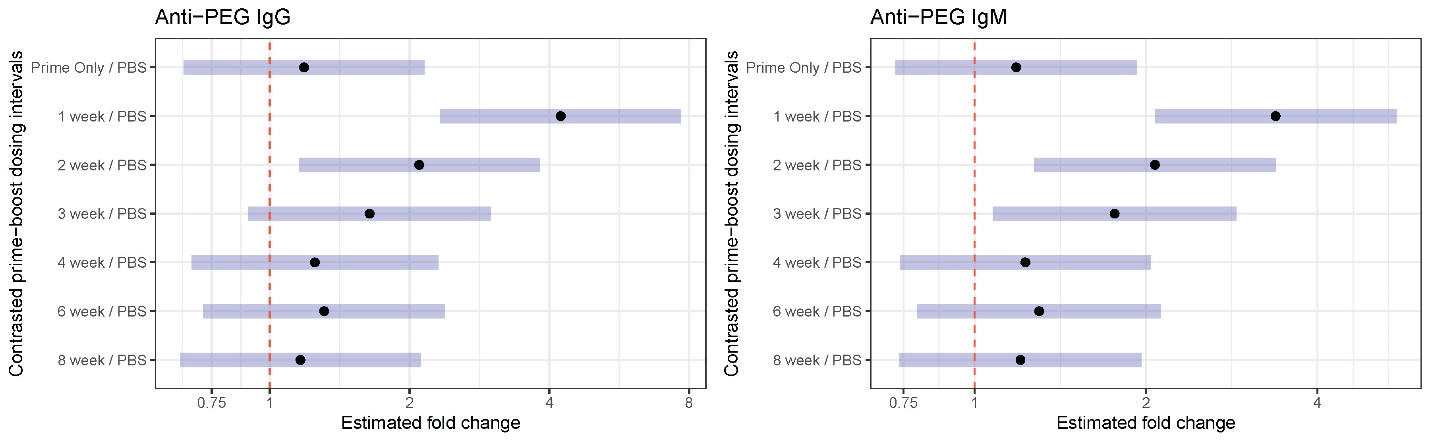
***

**3. Supplementary Tables**

### ***Supplementary Table 1. Statistically Significant Comparisons of S2-P IgG Antibody Titers in Mice Between Different mRNA-1273 Dosing Intervals.***

| **Pairwise comparison^a^** | **Fold change** | **Day post-dose 2 (approximate weeks post dose 2)** | **Adjusted  *P*-value^b^** |
| --- | --- | --- | --- |
| **mRNA-1273 1-µg dose level** | | | |
| 2 / 1 | 7.962 | 0 (pre-dose 2) | <1E-06 |
| 3 / 1 | 5.310 | 0 (pre-dose 2) | <1E-06 |
| 4 / 1 | 3.476 | 0 (pre-dose 2) | <1E-04 |
| 4 / 2 | 0.436 | 0 (pre-dose 2) | 0.014 |
| 6 / 1 | 2.362 | 0 (pre-dose 2) | 0.012 |
| 6 / 2 | 0.297 | 0 (pre-dose 2) | <1E-04 |
| 6 / 3 | 0.445 | 0 (pre-dose 2) | 0.022 |
| 8 / 1 | 3.544 | 0 (pre-dose 2) | <1E-04 |
| 8 / 2 | 0.445 | 0 (pre-dose 2) | 0.020 |
| 2 / 1 | 3.263 | 7 (1) | <1E-05 |
| 3 / 1 | 3.486 | 7 (1) | <1E-06 |
| 4 / 1 | 6.834 | 7 (1) | <1E-06 |
| 4 / 2 | 2.095 | 7 (1) | 0.015 |
| 4 / 3 | 1.960 | 7 (1) | 0.046 |
| 6 / 1 | 10.110 | 7 (1) | <1E-06 |
| 6 / 2 | 3.099 | 7 (1) | <1E-04 |
| 6 / 3 | 2.900 | 7 (1) | <1E-03 |
| 8 / 1 | 17.271 | 7 (1) | <1E-06 |
| 8 / 2 | 5.293 | 7 (1) | <1E-06 |
| 8 / 3 | 4.954 | 7 (1) | <1E-06 |
| 8 / 4 | 2.527 | 7 (1) | 0.003 |
| 2 / 1 | 2.050 | 16 (2) | 0.017 |
| 3 / 1 | 3.200 | 16 (2) | <1E-05 |
| 4 / 1 | 7.772 | 16 (2) | <1E-06 |
| 4 / 2 | 3.791 | 16 (2) | <1E-06 |
| 4 / 3 | 2.429 | 16 (2) | 0.003 |
| 6 / 1 | 12.382 | 16 (2) | <1E-06 |
| 6 / 2 | 6.040 | 16 (2) | <1E-06 |
| 6 / 3 | 3.870 | 16 (2) | <1E-05 |
| 8 / 1 | 18.423 | 16 (2) | <1E-06 |
| 8 / 2 | 8.987 | 16 (2) | <1E-06 |
| 8 / 3 | 5.758 | 16 (2) | <1E-06 |
| 8 / 4 | 2.371 | 16 (2) | 0.009 |
| 3 / 1 | 3.492 | 30 (4) | <1E-05 |
| 4 / 1 | 5.972 | 30 (4) | <1E-06 |
| 4 / 2 | 3.153 | 30 (4) | <1E-04 |
| 6 / 1 | 8.435 | 30 (4) | <1E-06 |
| 6 / 2 | 4.453 | 30 (4) | <1E-06 |
| 6 / 3 | 2.416 | 30 (4) | 0.008 |
| 8 / 1 | 10.847 | 30 (4) | <1E-06 |
| 8 / 2 | 5.726 | 30 (4) | <1E-06 |
| 8 / 3 | 3.107 | 30 (4) | <1E-03 |
| 4 / 1 | 5.635 | 58 (8) | <1E-06 |
| 4 / 2 | 3.288 | 58 (8) | <1E-04 |
| 4 / 3 | 3.100 | 58 (8) | <1E-03 |
| 6 / 1 | 7.882 | 58 (8) | <1E-06 |
| 6 / 2 | 4.599 | 58 (8) | <1E-06 |
| 6 / 3 | 4.337 | 58 (8) | <1E-05 |
| 8 / 1 | 8.157 | 58 (8) | <1E-06 |
| 8 / 2 | 4.759 | 58 (8) | <1E-06 |
| 8 / 3 | 4.488 | 58 (8) | <1E-06 |
| 4 / 1 | 3.955 | 86 (12) | <1E-04 |
| 4 / 2 | 2.575 | 86 (12) | 0.008 |
| 4 / 3 | 2.310 | 86 (12) | 0.034 |
| 6 / 1 | 8.233 | 86 (12) | <1E-06 |
| 6 / 2 | 5.361 | 86 (12) | <1E-06 |
| 6 / 3 | 4.808 | 86 (12) | <1E-06 |
| 8 / 1 | 9.791 | 86 (12) | <1E-06 |
| 8 / 2 | 6.375 | 86 (12) | <1E-06 |
| 8 / 3 | 5.718 | 86 (12) | <1E-06 |
| 8 / 4 | 2.476 | 86 (12) | 0.016 |
| 4 / 1 | 5.113 | 114 (16) | <1E-05 |
| 4 / 2 | 3.327 | 114 (16) | <1E-03 |
| 4 / 3 | 2.485 | 114 (16) | 0.028 |
| 6 / 1 | 12.221 | 114 (16) | <1E-06 |
| 6 / 2 | 7.953 | 114 (16) | <1E-06 |
| 6 / 3 | 5.941 | 114 (16) | <1E-06 |
| 6 / 4 | 2.390 | 114 (16) | 0.042 |
| 8 / 1 | 9.725 | 114 (16) | <1E-06 |
| 8 / 2 | 6.328 | 114 (16) | <1E-06 |
| 8 / 3 | 4.728 | 114 (16) | <1E-05 |
| 2 / 1 | 2.546 | 142 (20) | 0.034 |
| 3 / 1 | 2.952 | 142 (20) | 0.011 |
| 4 / 1 | 6.440 | 142 (20) | <1E-06 |
| 4 / 2 | 2.530 | 142 (20) | 0.039 |
| 6 / 1 | 15.759 | 142 (20) | <1E-06 |
| 6 / 2 | 6.190 | 142 (20) | <1E-06 |
| 6 / 3 | 5.338 | 142 (20) | <1E-05 |
| 8 / 1 | 13.152 | 142 (20) | <1E-06 |
| 8 / 2 | 5.166 | 142 (20) | <1E-05 |
| 8 / 3 | 4.455 | 142 (20) | <1E-04 |
| 3 / 1 | 4.434 | 169 (24) | <1E-03 |
| 4 / 1 | 12.022 | 169 (24) | <1E-06 |
| 4 / 2 | 4.642 | 169 (24) | <1E-03 |
| 6 / 1 | 23.760 | 169 (24) | <1E-06 |
| 6 / 2 | 9.174 | 169 (24) | <1E-06 |
| 6 / 3 | 5.358 | 169 (24) | <1E-04 |
| 8 / 1 | 22.154 | 169 (24) | <1E-06 |
| 8 / 2 | 8.554 | 169 (24) | <1E-06 |
| 8 / 3 | 4.996 | 169 (24) | <1E-03 |
| **mRNA-1273 10-µg dose level** | | | |
| 2 / 1 | 16.076 | 0 (pre-dose 2) | <1E-06 |
| 3 / 1 | 9.865 | 0 (pre-dose 2) | <1E-06 |
| 4 / 1 | 9.499 | 0 (pre-dose 2) | <1E-06 |
| 6 / 1 | 6.516 | 0 (pre-dose 2) | <1E-06 |
| 6 / 2 | 0.405 | 0 (pre-dose 2) | 0.006 |
| 8 / 1 | 8.375 | 0 (pre-dose 2) | <1E-06 |
| 2 / 1 | 6.524 | 7 (1) | <1E-06 |
| 3 / 1 | 6.489 | 7 (1) | <1E-06 |
| 4 / 1 | 18.040 | 7 (1) | <1E-06 |
| 4 / 2 | 2.765 | 7 (1) | <1E-03 |
| 4 / 3 | 2.780 | 7 (1) | <1E-03 |
| 6 / 1 | 26.720 | 7 (1) | <1E-06 |
| 6 / 2 | 4.095 | 7 (1) | <1E-06 |
| 6 / 3 | 4.118 | 7 (1) | <1E-06 |
| 8 / 1 | 39.665 | 7 (1) | <1E-06 |
| 8 / 2 | 6.080 | 7 (1) | <1E-06 |
| 8 / 3 | 6.112 | 7 (1) | <1E-06 |
| 8 / 4 | 2.199 | 7 (1) | 0.019 |
| 2 / 1 | 4.055 | 16 (2) | <1E-06 |
| 3 / 1 | 5.969 | 16 (2) | <1E-06 |
| 4 / 1 | 19.728 | 16 (2) | <1E-06 |
| 4 / 2 | 4.865 | 16 (2) | <1E-06 |
| 4 / 3 | 3.305 | 16 (2) | <1E-05 |
| 6 / 1 | 31.177 | 16 (2) | <1E-06 |
| 6 / 2 | 7.688 | 16 (2) | <1E-06 |
| 6 / 3 | 5.223 | 16 (2) | <1E-06 |
| 8 / 1 | 40.978 | 16 (2) | <1E-06 |
| 8 / 2 | 10.104 | 16 (2) | <1E-06 |
| 8 / 3 | 6.865 | 16 (2) | <1E-06 |
| 8 / 4 | 2.077 | 16 (2) | 0.044 |
| 2 / 1 | 3.685 | 30 (4) | <1E-05 |
| 3 / 1 | 6.536 | 30 (4) | <1E-06 |
| 4 / 1 | 14.267 | 30 (4) | <1E-06 |
| 4 / 2 | 3.872 | 30 (4) | <1E-05 |
| 4 / 3 | 2.183 | 30 (4) | 0.026 |
| 6 / 1 | 19.700 | 30 (4) | <1E-06 |
| 6 / 2 | 5.346 | 30 (4) | <1E-06 |
| 6 / 3 | 3.014 | 30 (4) | <1E-03 |
| 8 / 1 | 22.955 | 30 (4) | <1E-06 |
| 8 / 2 | 6.229 | 30 (4) | <1E-06 |
| 8 / 3 | 3.512 | 30 (4) | <1E-04 |
| 2 / 1 | 3.224 | 58 (8) | <1E-04 |
| 3 / 1 | 3.425 | 58 (8) | <1E-04 |
| 4 / 1 | 11.919 | 58 (8) | <1E-06 |
| 4 / 2 | 3.697 | 58 (8) | <1E-04 |
| 4 / 3 | 3.480 | 58 (8) | <1E-04 |
| 6 / 1 | 15.834 | 58 (8) | <1E-06 |
| 6 / 2 | 4.911 | 58 (8) | <1E-06 |
| 6 / 3 | 4.624 | 58 (8) | <1E-06 |
| 8 / 1 | 15.625 | 58 (8) | <1E-06 |
| 8 / 2 | 4.846 | 58 (8) | <1E-06 |
| 8 / 3 | 4.562 | 58 (8) | <1E-06 |
| 2 / 1 | 2.794 | 86 (12) | 0.002 |
| 3 / 1 | 3.249 | 86 (12) | <1E-03 |
| 4 / 1 | 7.408 | 86 (12) | <1E-06 |
| 4 / 2 | 2.651 | 86 (12) | 0.005 |
| 4 / 3 | 2.280 | 86 (12) | 0.037 |
| 6 / 1 | 14.229 | 86 (12) | <1E-06 |
| 6 / 2 | 5.093 | 86 (12) | <1E-06 |
| 6 / 3 | 4.380 | 86 (12) | <1E-05 |
| 8 / 1 | 16.975 | 86 (12) | <1E-06 |
| 8 / 2 | 6.076 | 86 (12) | <1E-06 |
| 8 / 3 | 5.225 | 86 (12) | <1E-06 |
| 8 / 4 | 2.291 | 86 (12) | 0.038 |
| 2 / 1 | 2.703 | 114 (16) | 0.008 |
| 3 / 1 | 3.929 | 114 (16) | <1E-04 |
| 4 / 1 | 8.480 | 114 (16) | <1E-06 |
| 4 / 2 | 3.137 | 114 (16) | 0.001 |
| 6 / 1 | 18.170 | 114 (16) | <1E-06 |
| 6 / 2 | 6.722 | 114 (16) | <1E-06 |
| 6 / 3 | 4.625 | 114 (16) | <1E-05 |
| 8 / 1 | 15.261 | 114 (16) | <1E-06 |
| 8 / 2 | 5.646 | 114 (16) | <1E-05 |
| 8 / 3 | 3.885 | 114 (16) | <1E-04 |
| 2 / 1 | 4.330 | 142 (20) | <1E-04 |
| 3 / 1 | 5.676 | 142 (20) | <1E-05 |
| 4 / 1 | 9.459 | 142 (20) | <1E-06 |
| 6 / 1 | 20.155 | 142 (20) | <1E-06 |
| 6 / 2 | 4.654 | 142 (20) | <1E-04 |
| 6 / 3 | 3.551 | 142 (20) | 0.001 |
| 8 / 1 | 18.681 | 142 (20) | <1E-06 |
| 8 / 2 | 4.314 | 142 (20) | <1E-04 |
| 8 / 3 | 3.291 | 142 (20) | 0.003 |
| 2 / 1 | 4.265 | 169 (24) | <1E-03 |
| 3 / 1 | 8.581 | 169 (24) | <1E-06 |
| 4 / 1 | 15.704 | 169 (24) | <1E-06 |
| 4 / 2 | 3.682 | 169 (24) | 0.002 |
| 6 / 1 | 26.282 | 169 (24) | <1E-06 |
| 6 / 2 | 6.162 | 169 (24) | <1E-05 |
| 6 / 3 | 3.063 | 169 (24) | 0.016 |
| 8 / 1 | 28.584 | 169 (24) | <1E-06 |
| 8 / 2 | 6.702 | 169 (24) | <1E-06 |
| 8 / 3 | 3.331 | 169 (24) | 0.007 |

IgG, immunoglobulin G; mRNA, messenger RNA.

^a^Pairwise comparison X/Y refers to the statistical comparison of the fold change in S2-P IgG antibody titer at the X-week dosing interval group compared to the Y-week dosing interval group.

^b^P-values are based on a 2-sided t test with a multivariate t adjustment. Though all pairwise comparisons were performed, only those that met a significance level of 0.05 are shown.

### Supplementary Table 2. Statistical Comparisons of Antibody Fc-effector Function Responses Between mRNA-1273 Dosing Intervals.

| **Pairwise comparison^a^** | **Adjusted *P-*value^b^ (mRNA-1273 1-µg dose level)** | **Adjusted *P*-value^b^ (mRNA-1273 10-µg dose level)** |
| --- | --- | --- |
| 2 / 1 | <1E-06 | <1E-06 |
| 3 / 1 | <1E-06 | <1E-06 |
| 3 / 2 | 1.000 | 1.000 |
| 4 / 1 | <1E-06 | <1E-06 |
| 4 / 2 | <1E-06 | <1E-06 |
| 4 / 3 | <1E-06 | <1E-06 |
| 6 / 1 | <1E-06 | <1E-06 |
| 6 / 2 | <1E-06 | <1E-06 |
| 6 / 3 | <1E-06 | <1E-06 |
| 6 / 4 | <1E-06 | <1E-06 |
| 8 / 1 | <1E-06 | <1E-06 |
| 8 / 2 | <1E-06 | <1E-06 |
| 8 / 3 | <1E-06 | <1E-06 |
| 8 / 4 | <1E-06 | <1E-06 |
| 8 / 6 | 0.677 | 0.095 |

ADCC, antibody-dependent cellular cytotoxicity; mRNA, messenger RNA.

^a^Pairwise comparison X/Y refers to the statistical comparison of the log_2_ fold change in ADCC activity for the X-week dosing interval group over the Y-week dosing interval group.

^b^P-values were calculated using a 1-sided Bayesian maximum a priori test with Bonferroni adjustment to determine whether the ADCC activity in the X-week dosing interval group was greater than in the Y-week dosing interval group.

### Supplementary Table 3. Statistically Significant Comparisons of S2-P-specific ASCs 1 Week Following Dose 2 Between mRNA-1273 (10 µg) Dosing Intervals.

| **Pairwise comparison^a^** | **Fold change** | **Adjusted *P*-value^b^** |
| --- | --- | --- |
| 0 / 3 | 0.440 | 0.013 |
| 0 / 4 | 0.337 | <1E-03 |
| 0 / 6 | 0.237 | <1E-05 |
| 0 / 8 | 0.204 | <1E-06 |
| 1 / 3 | 0.333 | <1E-03 |
| 1 / 4 | 0.255 | <1E-04 |
| 1 / 6 | 0.179 | <1E-06 |
| 1 / 8 | 0.154 | <1E-06 |
| 2 / 4 | 0.480 | 0.012 |
| 2 / 6 | 0.337 | <1E-04 |
| 2 / 8 | 0.290 | <1E-05 |
| 3 / 6 | 0.537 | 0.012 |
| 3 / 8 | 0.463 | <1E-03 |
| 4 / 8 | 0.604 | 0.037 |

ASC, antibody-secreting cell; mRNA, messenger RNA.

^a^Pairwise comparison X/Y refers to the statistical comparison of the fold change in spike-specific antibody secreting cells at the X-week dosing interval group compared to the Y-week dosing interval group.

^b^*P*-values are based on a 2-sided *t* test with a multivariate *t* adjustment. Though all pairwise comparisons were performed, only those that met a significance level of 0.05 are shown.

### Supplementary Table 4. Statistically Significant Comparisons of S2-P–specific LLPCs 4 Weeks Following Dose 2 and 24 Weeks Following Dose 2 Between mRNA-1273 (10 µg) Dosing Intervals.

| **Days post dose 2**  **(approximate weeks post-dose 2)** | **Pairwise comparison** | **Fold change** | **Adjusted *P*-value** |
| --- | --- | --- | --- |
| 30 (4) | 0 / 4 | 0.102 | <1E-06 |
| 30 (4) | 0 / 6 | 0.094 | <1E-06 |
| 30 (4) | 0 / 8 | 0.160 | <1E-04 |
| 30 (4) | 1 / 2 | 0.308 | 0.016 |
| 30 (4) | 1 / 4 | 0.083 | <1E-06 |
| 30 (4) | 1 / 6 | 0.077 | <1E-06 |
| 30 (4) | 1 / 8 | 0.131 | <1E-06 |
| 30 (4) | 2 / 4 | 0.271 | <1E-04 |
| 30 (4) | 2 / 6 | 0.249 | <1E-05 |
| 30 (4) | 2 / 8 | 0.426 | 0.016 |
| 30 (4) | 3 / 4 | 0.239 | <1E-05 |
| 30 (4) | 3 / 6 | 0.220 | <1E-05 |
| 30 (4) | 3 / 8 | 0.376 | 0.008 |
| 169 (24) | 0 / 4 | 0.184 | <1E-04 |
| 169 (24) | 0 / 6 | 0.101 | <1E-06 |
| 169 (24) | 0 / 8 | 0.070 | <1E-06 |
| 169 (24) | 1 / 4 | 0.127 | <1E-05 |
| 169 (24) | 1 / 6 | 0.070 | <1E-06 |
| 169 (24) | 1 / 8 | 0.049 | <1E-06 |
| 169 (24) | 2 / 4 | 0.291 | <1E-03 |
| 169 (24) | 2 / 6 | 0.160 | <1E-06 |
| 169 (24) | 2 / 8 | 0.112 | <1E-06 |
| 169 (24) | 3 / 4 | 0.268 | 0.001 |
| 169 (24) | 3 / 6 | 0.148 | <1E-06 |
| 169 (24) | 3 / 8 | 0.103 | <1E-06 |
| 169 (24) | 4 / 8 | 0.384 | <1E-03 |

LLPC, long-lived plasma cell; mRNA, messenger RNA.

^a^Pairwise comparison X/Y refers to the statistical comparison of the fold change in spike-specific LLPCs at the X-week dosing interval group compared to the Y-week dosing interval group.

^b^P-values are based on a 2-sided t test with a multivariate t adjustment. Though all pairwise comparisons were performed, only those that met a significance level of 0.05 are shown.

### Supplementary Table 5. Statistically Significant Comparisons of Percentage of SARS-CoV-2 Spike-specific IFNγ, IL-2, and TNFα Producing CD4+ T Cells Through 24 Weeks Following Dose 2.

| **Pairwise comparison^s^** | **Fold change** | **Days post dose 2**  **(approximate weeks post-dose 2)** | **Cytokine** | **Coating** | **Adjusted *P*-value^b^** |
| --- | --- | --- | --- | --- | --- |
| 1 / 3 | 0.396 | 7 (1) | IFNγ | S1 | <1E-04 |
| 1 / 6 | 0.496 | 7 (1) | IFNγ | S1 | 0.017 |
| 3 / 4 | 1.905 | 7 (1) | IFNγ | S1 | 0.010 |
| 3 / 8 | 2.059 | 7 (1) | IFNγ | S1 | 0.001 |
| 0 / 1 | 6.025 | 86 (12) | IFNγ | S1 | 0.003 |
| 0 / 6 | 4.155 | 86 (12) | IFNγ | S1 | 0.016 |
| 0 / 8 | 5.395 | 86 (12) | IFNγ | S1 | <1E-03 |
| 0 / 2 | 0.190 | 169 (24) | IFNγ | S1 | 0.005 |
| 0 / 3 | 0.128 | 169 (24) | IFNγ | S1 | <1E-06 |
| 0 / 4 | 0.337 | 169 (24) | IFNγ | S1 | 0.015 |
| 0 / 6 | 0.191 | 169 (24) | IFNγ | S1 | <1E-04 |
| 0 / 8 | 0.136 | 169 (24) | IFNγ | S1 | <1E-05 |
| 0 / 1 | 2.955 | 7 (1) | IL-2 | S1 | 0.002 |
| 1 / 3 | 0.320 | 7 (1) | IL-2 | S1 | <1E-06 |
| 3 / 8 | 2.174 | 7 (1) | IL-2 | S1 | <1E-03 |
| 1 / 2 | 0.449 | 86 (12) | IL-2 | S1 | <1E-03 |
| 1 / 3 | 0.459 | 86 (12) | IL-2 | S1 | <1E-04 |
| 1 / 4 | 0.594 | 86 (12) | IL-2 | S1 | 0.025 |
| 1 / 6 | 0.594 | 86 (12) | IL-2 | S1 | 0.004 |
| 1 / 3 | 0.415 | 169 (24) | IL-2 | S1 | 0.008 |
| 0 / 1 | 0.433 | 7 (1) | TNFα | S1 | <1E-03 |
| 0 / 8 | 0.494 | 7 (1) | TNFα | S1 | 0.009 |
| 1 / 2 | 0.539 | 7 (1) | TNFα | S1 | 0.002 |
| 1 / 3 | 0.501 | 7 (1) | TNFα | S1 | 0.002 |
| 1 / 4 | 0.600 | 7 (1) | TNFα | S1 | 0.042 |
| 1 / 6 | 0.535 | 7 (1) | TNFα | S1 | 0.029 |
| 2 / 8 | 0.465 | 7 (1) | TNFα | S1 | 0.002 |
| 3 / 8 | 0.399 | 7 (1) | TNFα | S1 | <1E-04 |
| 4 / 8 | 0.555 | 7 (1) | TNFα | S1 | 0.019 |
| 6 / 8 | 0.483 | 7 (1) | TNFα | S1 | <1E-03 |
| 1 / 2 | 0.414 | 30 (4) | TNFα | S1 | <1E-05 |
| 1 / 2 | 0.401 | 86 (12) | TNFα | S1 | 0.010 |
| 1 / 3 | 0.465 | 86 (12) | TNFα | S1 | 0.010 |
| 1 / 4 | 2.000 | 86 (12) | TNFα | S1 | 0.005 |
| 1 / 6 | 1.768 | 86 (12) | TNFα | S1 | 0.016 |
| 1 / 8 | 0.420 | 86 (12) | TNFα | S1 | <1E-03 |
| 0 / 4 | 0.324 | 169 (24) | TNFα | S1 | 0.015 |
| 0 / 6 | 0.166 | 169 (24) | TNFα | S1 | <1E-06 |
| 0 / 8 | 0.428 | 169 (24) | TNFα | S1 | 0.023 |
| 1 / 4 | 0.287 | 169 (24) | TNFα | S1 | <1E-05 |
| 1 / 6 | 0.371 | 169 (24) | TNFα | S1 | 0.008 |
| 1 / 8 | 0.396 | 169 (24) | TNFα | S1 | <1E-04 |
| 3 / 8 | 2.580 | 169 (24) | TNFα | S1 | 0.006 |
| 0 / 3 | 0.486 | 7 (1) | IFNγ | S2 | 0.014 |
| 3 / 8 | 0.572 | 7 (1) | IFNγ | S2 | 0.013 |
| 3 / 8 | 0.411 | 86 (12) | IFNγ | S2 | <1E-06 |
| 0 / 1 | 0.567 | 169 (24) | IFNγ | S2 | <1E-03 |
| 0 / 2 | 0.408 | 169 (24) | IFNγ | S2 | <1E-06 |
| 0 / 3 | 0.615 | 169 (24) | IFNγ | S2 | 0.007 |
| 0 / 4 | 1.940 | 169 (24) | IFNγ | S2 | <1E-05 |
| 0 / 6 | 1.510 | 169 (24) | IFNγ | S2 | 0.012 |
| 0 / 8 | 1.868 | 169 (24) | IFNγ | S2 | <1E-06 |
| 1 / 3 | 0.576 | 169 (24) | IFNγ | S2 | 0.034 |
| 3 / 4 | 0.433 | 169 (24) | IFNγ | S2 | <1E-03 |
| 0 / 3 | 0.575 | 7 (1) | IL-2 | S2 | 0.047 |
| 0 / 2 | 0.562 | 86 (12) | IL-2 | S2 | 0.021 |
| 0 / 3 | 0.444 | 86 (12) | IL-2 | S2 | <1E-03 |
| 1 / 2 | 0.600 | 86 (12) | IL-2 | S2 | 0.007 |
| 1 / 3 | 1.849 | 86 (12) | IL-2 | S2 | <1E-04 |
| 1 / 6 | 2.630 | 86 (12) | IL-2 | S2 | <1E-06 |
| 3 / 4 | 1.948 | 86 (12) | IL-2 | S2 | <1E-06 |
| 3 / 6 | 2.168 | 86 (12) | IL-2 | S2 | <1E-03 |
| 3 / 8 | 0.479 | 86 (12) | IL-2 | S2 | 0.006 |
| 0 / 2 | 0.589 | 169 (24) | IL-2 | S2 | 0.009 |
| 0 / 3 | 0.516 | 169 (24) | IL-2 | S2 | 0.002 |
| 0 / 4 | 0.551 | 169 (24) | IL-2 | S2 | <1E-03 |
| 0 / 6 | 0.416 | 169 (24) | IL-2 | S2 | <1E-06 |
| 0 / 2 | 0.544 | 7 (1) | TNFα | S2 | <1E-03 |
| 0 / 3 | 0.559 | 7 (1) | TNFα | S2 | <1E-03 |
| 0 / 4 | 0.667 | 7 (1) | TNFα | S2 | 0.046 |
| 0 / 6 | 1.601 | 7 (1) | TNFα | S2 | 0.015 |
| 2 / 8 | 0.559 | 7 (1) | TNFα | S2 | 0.046 |
| 3 / 8 | 0.509 | 7 (1) | TNFα | S2 | 0.001 |
| 4 / 8 | 0.510 | 7 (1) | TNFα | S2 | 0.018 |
| 6 / 8 | 0.610 | 7 (1) | TNFα | S2 | 0.024 |
| 1 / 2 | 0.555 | 30 (4) | TNFα | S2 | <1E-04 |
| 1 / 3 | 0.557 | 30 (4) | TNFα | S2 | 0.008 |
| 0 / 3 | 3.376 | 86 (12) | TNFα | S2 | 0.040 |
| 1 / 2 | 2.365 | 86 (12) | TNFα | S2 | <1E-05 |
| 1 / 3 | 2.802 | 86 (12) | TNFα | S2 | <1E-04 |
| 1 / 4 | 8.379 | 86 (12) | TNFα | S2 | <1E-06 |
| 1 / 6 | 6.245 | 86 (12) | TNFα | S2 | <1E-06 |
| 1 / 8 | 6.930 | 86 (12) | TNFα | S2 | <1E-06 |
| 3 / 8 | 5.411 | 86 (12) | TNFα | S2 | <1E-06 |
| 0 / 2 | 0.209 | 169 (24) | TNFα | S2 | <1E-04 |
| 0 / 4 | 0.281 | 169 (24) | TNFα | S2 | 0.003 |
| 0 / 6 | 3.952 | 169 (24) | TNFα | S2 | <1E-04 |
| 0 / 8 | 3.086 | 169 (24) | TNFα | S2 | 0.007 |
| 1 / 2 | 8.655 | 169 (24) | TNFα | S2 | 0.002 |
| 1 / 4 | 10.483 | 169 (24) | TNFα | S2 | <1E-03 |
| 1 / 6 | 10.574 | 169 (24) | TNFα | S2 | <1E-04 |
| 1 / 8 | 5.081 | 169 (24) | TNFα | S2 | <1E-06 |

IFNγ, interferon γ; IL-2, interleukin-2; S1, subunit 1; S2, subunit 2, SARS-CoV-2, severe acute respiratory syndrome coronavirus 2; TNFα, tumor necrosis factor α.

^a^Pairwise comparison X/Y refers to the statistical comparison of the fold change in percentage of SARS-CoV-2 spike-specific IFNγ producing CD4+ T cells at the X-week dosing interval group compared to Y-week dosing interval group.

^b^*P*-values are based on a 2-sided *t* test with multivariate *t* adjustment. Though all pairwise comparisons were performed, only those that met a significance level of 0.05 are shown.

### Supplementary Table 6. Statistically Significant Comparisons of Proportions of Polyfunctional CD4+ or CD8+ T Cells Between mRNA-1273 Dosing Interval Groups.

| **Pairwise comparison^a^** | **Odds ratio^b^** | **Days post dose 2**  **(approximate weeks post dose 2)** | **Day** | **Number positive** | **Adjusted  *P*-value^c^** |
| --- | --- | --- | --- | --- | --- |
| **CD4+ T cells** | | | | | |
| 6 / 4 | 0.524 | 30 (4) | 87 | 1 | 0.048 |
| 3 / 2 | 0.39 | 86 (12) | 143 | 1 | 0.046 |
| 4 / 2 | 0.338 | 86 (12) | 143 | 1 | 0.013 |
| 6 / 2 | 0.38 | 86 (12) | 143 | 1 | 0.031 |
| 8 / 2 | 0.344 | 86 (12) | 143 | 1 | 0.015 |
| 3 / 0 | 1.493 | 7 (1) | 64 | 2 | 0.002 |
| 3 / 1 | 1.537 | 7 (1) | 64 | 2 | <1E-04 |
| 8 / 2 | 1.768 | 7 (1) | 64 | 2 | <1E-06 |
| 8 / 3 | 1.819 | 7 (1) | 64 | 2 | <1E-06 |
| 2 / 1 | 1.324 | 30 (4) | 87 | 2 | 0.039 |
| 3 / 1 | 0.728 | 30 (4) | 87 | 2 | 0.003 |
| 8 / 1 | 1.407 | 30 (4) | 87 | 2 | 0.014 |
| 3 / 0 | 1.448 | 86 (12) | 143 | 2 | <1E-03 |
| 3 / 1 | 0.696 | 86 (12) | 143 | 2 | <1E-03 |
| 4 / 3 | 1.493 | 86 (12) | 143 | 2 | 0.003 |
| 6 / 1 | 1.537 | 86 (12) | 143 | 2 | <1E-04 |
| 8 / 3 | 1.768 | 86 (12) | 143 | 2 | <1E-06 |
| 3 / 0 | 1.819 | 7 (1) | 64 | 3 | <1E-06 |
| 6 / 0 | 1.324 | 7 (1) | 64 | 3 | 0.039 |
| 8 / 1 | 0.728 | 7 (1) | 64 | 3 | 0.003 |
| 8 / 3 | 1.407 | 7 (1) | 64 | 3 | 0.013 |
| 8 / 6 | 1.448 | 7 (1) | 64 | 3 | <1E-03 |
| 2 / 1 | 0.696 | 30 (4) | 87 | 3 | <1E-03 |
| 3 / 0 | 1.493 | 30 (4) | 87 | 3 | 0.003 |
| 3 / 1 | 1.537 | 30 (4) | 87 | 3 | <1E-04 |
| 4 / 1 | 1.768 | 30 (4) | 87 | 3 | <1E-06 |
| 6 / 1 | 1.819 | 30 (4) | 87 | 3 | <1E-06 |
| 3 / 0 | 1.324 | 86 (12) | 143 | 3 | 0.039 |
| 3 / 1 | 0.728 | 86 (12) | 143 | 3 | 0.003 |
| 4 / 3 | 1.407 | 86 (12) | 143 | 3 | 0.013 |
| 6 / 3 | 1.448 | 86 (12) | 143 | 3 | <1E-03 |
| 8 / 3 | 0.696 | 86 (12) | 143 | 3 | <1E-03 |
| 3 / 0 | 1.493 | 169 (24) | 226 | 3 | 0.003 |
| 6 / 0 | 1.537 | 169 (24) | 226 | 3 | <1E-04 |
| **CD8+ T cells** | | | | | |
| 2 / 0 | 2.085 | 7 (1) | 64 | 1 | 0.029 |
| 2 / 1 | 2.321 | 7 (1) | 64 | 1 | <1E-03 |
| 3 / 1 | 2.018 | 7 (1) | 64 | 1 | 0.002 |
| 4 / 1 | 2.279 | 7 (1) | 64 | 1 | 0.006 |
| 6 / 0 | 4.103 | 7 (1) | 64 | 1 | <1E-06 |
| 6 / 1 | 4.568 | 7 (1) | 64 | 1 | <1E-06 |
| 6 / 2 | 1.968 | 7 (1) | 64 | 1 | 0.005 |
| 6 / 3 | 2.263 | 7 (1) | 64 | 1 | <1E-04 |
| 6 / 4 | 2.004 | 7 (1) | 64 | 1 | 0.029 |
| 8 / 0 | 2.925 | 7 (1) | 64 | 1 | <1E-04 |
| 8 / 1 | 3.256 | 7 (1) | 64 | 1 | <1E-06 |
| 6 / 1 | 2.031 | 30 (4) | 87 | 1 | 0.022 |
| 6 / 3 | 2.576 | 30 (4) | 87 | 1 | <1E-03 |
| 8 / 1 | 1.985 | 30 (4) | 87 | 1 | 0.021 |
| 8 / 3 | 2.517 | 30 (4) | 87 | 1 | <1E-03 |
| 2 / 1 | 2.235 | 86 (12) | 143 | 1 | 0.030 |
| 4 / 1 | 2.83 | 86 (12) | 143 | 1 | 0.003 |
| 6 / 0 | 3.886 | 86 (12) | 143 | 1 | <1E-03 |
| 6 / 1 | 4.686 | 86 (12) | 143 | 1 | <1E-06 |
| 6 / 2 | 2.097 | 86 (12) | 143 | 1 | 0.039 |
| 6 / 3 | 2.405 | 86 (12) | 143 | 1 | 0.001 |
| 8 / 0 | 3.392 | 86 (12) | 143 | 1 | 0.001 |
| 8 / 1 | 4.09 | 86 (12) | 143 | 1 | <1E-06 |
| 8 / 3 | 2.099 | 86 (12) | 143 | 1 | 0.012 |
| 3 / 1 | 2.423 | 169 (24) | 226 | 1 | 0.029 |
| 4 / 1 | 2.727 | 169 (24) | 226 | 1 | 0.038 |
| 6 / 0 | 3.944 | 169 (24) | 226 | 1 | <1E-03 |
| 6 / 1 | 5.15 | 169 (24) | 226 | 1 | <1E-06 |
| 8 / 0 | 3.939 | 169 (24) | 226 | 1 | <1E-03 |
| 8 / 1 | 5.143 | 169 (24) | 226 | 1 | <1E-06 |
| 8 / 3 | 2.123 | 169 (24) | 226 | 1 | 0.040 |
| 6 / 0 | 2.067 | 7 (1) | 64 | 2 | <1E-04 |
| 6 / 1 | 1.946 | 7 (1) | 64 | 2 | <1E-04 |
| 6 / 0 | 1.896 | 30 (4) | 87 | 2 | <1E-05 |
| 6 / 1 | 1.785 | 30 (4) | 87 | 2 | <1E-04 |
| 8 / 0 | 2.47 | 30 (4) | 87 | 2 | <1E-06 |
| 8 / 1 | 2.326 | 30 (4) | 87 | 2 | <1E-06 |
| 2 / 1 | 4.562 | 86 (12) | 143 | 2 | <1E-06 |
| 3 / 0 | 4.295 | 86 (12) | 143 | 2 | <1E-06 |
| 3 / 1 | 2.207 | 86 (12) | 143 | 2 | <1E-05 |
| 4 / 0 | 2.406 | 86 (12) | 143 | 2 | <1E-06 |
| 4 / 1 | 1.847 | 86 (12) | 143 | 2 | <1E-03 |
| 6 / 0 | 3.353 | 86 (12) | 143 | 2 | <1E-06 |
| 6 / 1 | 3.157 | 86 (12) | 143 | 2 | <1E-06 |
| 6 / 2 | 1.622 | 86 (12) | 143 | 2 | 0.022 |
| 6 / 3 | 1.769 | 86 (12) | 143 | 2 | <1E-03 |
| 8 / 0 | 2.067 | 86 (12) | 143 | 2 | <1E-05 |
| 8 / 1 | 1.946 | 86 (12) | 143 | 2 | <1E-04 |
| 2 / 0 | 1.896 | 169 (24) | 226 | 2 | <1E-05 |
| 3 / 0 | 1.785 | 169 (24) | 226 | 2 | <1E-04 |
| 6 / 0 | 2.47 | 169 (24) | 226 | 2 | <1E-06 |
| 6 / 1 | 2.326 | 169 (24) | 226 | 2 | <1E-06 |
| 6 / 2 | 4.562 | 169 (24) | 226 | 2 | <1E-06 |
| 6 / 3 | 4.295 | 169 (24) | 226 | 2 | <1E-06 |
| 6 / 4 | 2.207 | 169 (24) | 226 | 2 | <1E-05 |
| 8 / 0 | 2.406 | 169 (24) | 226 | 2 | <1E-06 |
| 8 / 1 | 1.847 | 169 (24) | 226 | 2 | <1E-03 |
| 8 / 2 | 3.353 | 169 (24) | 226 | 2 | <1E-06 |
| 8 / 3 | 3.157 | 169 (24) | 226 | 2 | <1E-06 |
| 8 / 4 | 1.622 | 169 (24) | 226 | 2 | 0.022 |
| 8 / 1 | 1.769 | 30 (4) | 87 | 3 | <1E-03 |
| 8 / 3 | 2.067 | 30 (4) | 87 | 3 | <1E-05 |
| 2 / 1 | 1.946 | 86 (12) | 143 | 3 | <1E-04 |
| 3 / 1 | 1.896 | 86 (12) | 143 | 3 | <1E-05 |
| 4 / 1 | 1.785 | 86 (12) | 143 | 3 | <1E-04 |
| 6 / 0 | 2.47 | 86 (12) | 143 | 3 | <1E-06 |
| 6 / 1 | 2.326 | 86 (12) | 143 | 3 | <1E-06 |
| 6 / 2 | 4.562 | 86 (12) | 143 | 3 | <1E-06 |
| 6 / 3 | 4.295 | 86 (12) | 143 | 3 | <1E-06 |
| 8 / 1 | 2.207 | 86 (12) | 143 | 3 | <1E-05 |
| 4 / 0 | 2.406 | 169 (24) | 226 | 3 | <1E-06 |
| 4 / 1 | 1.847 | 169 (24) | 226 | 3 | <1E-03 |
| 4 / 3 | 3.353 | 169 (24) | 226 | 3 | <1E-06 |
| 6 / 0 | 3.157 | 169 (24) | 226 | 3 | <1E-06 |
| 6 / 1 | 1.622 | 169 (24) | 226 | 3 | 0.022 |
| 6 / 2 | 1.769 | 169 (24) | 226 | 3 | <1E-03 |
| 6 / 3 | 2.067 | 169 (24) | 226 | 3 | <1E-04 |
| 8 / 0 | 1.946 | 169 (24) | 226 | 3 | <1E-04 |
| 8 / 1 | 1.896 | 169 (24) | 226 | 3 | <1E-05 |
| 8 / 2 | 1.785 | 169 (24) | 226 | 3 | <1E-04 |
| 8 / 3 | 2.47 | 169 (24) | 226 | 3 | <1E-06 |

mRNA. messenger RNA; S1. subunit 1; S2. subunit 2.

Comparisons are broken down by days following dose 2. cell population. and single. dual. and triple expressors. For CD4+ and CD8+ T cells. only the S2 and S1 peptide pools were considered. respectively.

^a^Pairwise comparison X/Y refers to the statistical comparison of the log_2_ fold change in the odds of polyfunctionality for the X-week dosing interval group over the Y-week dosing interval group.

^b^An odds ratio greater (less) then 1 indicates group X showed a significant increase (decrease) relative to group Y.

^c^P-values are based on a 2-sided t test with multivariate t adjustment. Though all pairwise comparisons were performed. only those that met a significance level of 0.05 are shown.

### Supplementary Table 7. Statistically Significant Comparisons of Percentage of SARS-CoV-2 Spike-specific IFNγ. IL-2. and TNFα Producing CD8+ T Cells Through 24 Weeks Following Dose 2.

| **Pairwise comparison** | **Fold change** | **Days post dose 2 (approximate weeks post dose 2)** | **Cytokine** | **Coating** | **Adjusted *P*-value** |
| --- | --- | --- | --- | --- | --- |
| 0 / 2 | 0.462 | 7 (1) | IFNγ | S1 | <1E-04 |
| 0 / 3 | 0.484 | 7 (1) | IFNγ | S1 | 0.016 |
| 0 / 4 | 0.387 | 7 (1) | IFNγ | S1 | <1E-05 |
| 0 / 6 | 0.209 | 7 (1) | IFNγ | S1 | <1E-06 |
| 0 / 8 | 0.378 | 7 (1) | IFNγ | S1 | <1E-06 |
| 1 / 2 | 0.476 | 7 (1) | IFNγ | S1 | 0.011 |
| 1 / 4 | 0.399 | 7 (1) | IFNγ | S1 | 0.001 |
| 1 / 6 | 0.215 | 7 (1) | IFNγ | S1 | <1E-06 |
| 1 / 8 | 0.390 | 7 (1) | IFNγ | S1 | <1E-03 |
| 2 / 6 | 0.452 | 7 (1) | IFNγ | S1 | <1E-04 |
| 3 / 6 | 0.431 | 7 (1) | IFNγ | S1 | 0.003 |
| 4 / 6 | 0.539 | 7 (1) | IFNγ | S1 | 0.014 |
| 6 / 8 | 1.811 | 7 (1) | IFNγ | S1 | 0.004 |
| 0 / 6 | 0.288 | 30 (4) | IFNγ | S1 | 0.003 |
| 0 / 8 | 0.282 | 30 (4) | IFNγ | S1 | 0.004 |
| 1 / 6 | 0.313 | 30 (4) | IFNγ | S1 | <1E-04 |
| 1 / 8 | 0.307 | 30 (4) | IFNγ | S1 | <1E-03 |
| 3 / 6 | 0.342 | 30 (4) | IFNγ | S1 | <1E-04 |
| 3 / 8 | 0.335 | 30 (4) | IFNγ | S1 | <1E-03 |
| 0 / 4 | 0.345 | 86 (12) | IFNγ | S1 | <1E-03 |
| 0 / 6 | 0.231 | 86 (12) | IFNγ | S1 | <1E-04 |
| 0 / 8 | 0.334 | 86 (12) | IFNγ | S1 | 0.001 |
| 1 / 2 | 0.412 | 86 (12) | IFNγ | S1 | 0.003 |
| 1 / 3 | 0.482 | 86 (12) | IFNγ | S1 | 0.019 |
| 1 / 4 | 0.272 | 86 (12) | IFNγ | S1 | <1E-06 |
| 1 / 6 | 0.182 | 86 (12) | IFNγ | S1 | <1E-06 |
| 1 / 8 | 0.263 | 86 (12) | IFNγ | S1 | <1E-05 |
| 2 / 6 | 0.442 | 86 (12) | IFNγ | S1 | 0.004 |
| 3 / 4 | 0.563 | 86 (12) | IFNγ | S1 | <1E-03 |
| 3 / 6 | 0.378 | 86 (12) | IFNγ | S1 | <1E-04 |
| 3 / 8 | 0.545 | 86 (12) | IFNγ | S1 | 0.028 |
| 0 / 6 | 0.193 | 169 (24) | IFNγ | S1 | <1E-06 |
| 0 / 8 | 0.211 | 169 (24) | IFNγ | S1 | <1E-06 |
| 1 / 2 | 0.489 | 169 (24) | IFNγ | S1 | 0.042 |
| 1 / 3 | 0.518 | 169 (24) | IFNγ | S1 | 0.011 |
| 1 / 4 | 0.537 | 169 (24) | IFNγ | S1 | 0.047 |
| 1 / 6 | 0.213 | 169 (24) | IFNγ | S1 | <1E-06 |
| 1 / 8 | 0.233 | 169 (24) | IFNγ | S1 | <1E-06 |
| 2 / 6 | 0.435 | 169 (24) | IFNγ | S1 | 0.024 |
| 3 / 6 | 0.412 | 169 (24) | IFNγ | S1 | 0.001 |
| 3 / 8 | 0.449 | 169 (24) | IFNγ | S1 | 0.004 |
| 4 / 6 | 0.397 | 169 (24) | IFNγ | S1 | 0.002 |
| 4 / 8 | 0.433 | 169 (24) | IFNγ | S1 | 0.005 |
| 1 / 6 | 0.234 | 7 (1) | IL-2 | S1 | 0.009 |
| 3 / 6 | 0.380 | 7 (1) | IL-2 | S1 | 0.008 |
| 0 / 3 | 4.388 | 30 (4) | IL-2 | S1 | 0.032 |
| 1 / 3 | 3.124 | 30 (4) | IL-2 | S1 | <1E-03 |
| 3 / 4 | 0.208 | 30 (4) | IL-2 | S1 | 0.004 |
| 3 / 8 | 0.256 | 30 (4) | IL-2 | S1 | <1E-03 |
| 0 / 4 | 0.500 | 86 (12) | IL-2 | S1 | <1E-04 |
| 0 / 6 | 0.306 | 86 (12) | IL-2 | S1 | <1E-06 |
| 0 / 8 | 0.506 | 86 (12) | IL-2 | S1 | 0.001 |
| 1 / 2 | 0.425 | 86 (12) | IL-2 | S1 | 0.006 |
| 1 / 3 | 0.489 | 86 (12) | IL-2 | S1 | 0.010 |
| 1 / 4 | 0.316 | 86 (12) | IL-2 | S1 | <1E-06 |
| 1 / 6 | 0.194 | 86 (12) | IL-2 | S1 | <1E-06 |
| 1 / 8 | 0.320 | 86 (12) | IL-2 | S1 | <1E-05 |
| 2 / 6 | 0.456 | 86 (12) | IL-2 | S1 | 0.010 |
| 3 / 6 | 0.396 | 86 (12) | IL-2 | S1 | <1E-04 |
| 0 / 4 | 0.402 | 169 (24) | IL-2 | S1 | 0.048 |
| 0 / 6 | 0.275 | 169 (24) | IL-2 | S1 | <1E-03 |
| 0 / 8 | 0.282 | 169 (24) | IL-2 | S1 | <1E-03 |
| 1 / 4 | 0.387 | 169 (24) | IL-2 | S1 | 0.003 |
| 1 / 6 | 0.264 | 169 (24) | IL-2 | S1 | <1E-06 |
| 1 / 8 | 0.271 | 169 (24) | IL-2 | S1 | <1E-05 |
| 3 / 4 | 0.420 | 169 (24) | IL-2 | S1 | 0.002 |
| 3 / 6 | 0.286 | 169 (24) | IL-2 | S1 | <1E-06 |
| 3 / 8 | 0.294 | 169 (24) | IL-2 | S1 | <1E-06 |
| 0 / 2 | 0.630 | 7 (1) | TNFα | S1 | 0.012 |
| 0 / 4 | 0.511 | 7 (1) | TNFα | S1 | 0.008 |
| 0 / 6 | 0.334 | 7 (1) | TNFα | S1 | <1E-06 |
| 1 / 2 | 0.548 | 7 (1) | TNFα | S1 | 0.034 |
| 1 / 3 | 0.446 | 7 (1) | TNFα | S1 | 0.042 |
| 1 / 4 | 0.445 | 7 (1) | TNFα | S1 | 0.011 |
| 1 / 6 | 0.291 | 7 (1) | TNFα | S1 | <1E-05 |
| 2 / 6 | 0.531 | 7 (1) | TNFα | S1 | 0.010 |
| 2 / 8 | 1.798 | 7 (1) | TNFα | S1 | 0.002 |
| 3 / 8 | 2.207 | 7 (1) | TNFα | S1 | 0.014 |
| 4 / 8 | 2.216 | 7 (1) | TNFα | S1 | 0.002 |
| 6 / 8 | 3.386 | 7 (1) | TNFα | S1 | <1E-06 |
| 0 / 2 | 0.235 | 30 (4) | TNFα | S1 | 0.009 |
| 0 / 6 | 0.186 | 30 (4) | TNFα | S1 | 0.001 |
| 0 / 8 | 0.178 | 30 (4) | TNFα | S1 | <1E-03 |
| 1 / 8 | 0.391 | 30 (4) | TNFα | S1 | 0.026 |
| 3 / 6 | 0.390 | 30 (4) | TNFα | S1 | 0.034 |
| 3 / 8 | 0.373 | 30 (4) | TNFα | S1 | 0.003 |
| 0 / 4 | 0.333 | 86 (12) | TNFα | S1 | <1E-04 |
| 0 / 6 | 0.232 | 86 (12) | TNFα | S1 | <1E-05 |
| 0 / 8 | 0.367 | 86 (12) | TNFα | S1 | 0.001 |
| 1 / 2 | 0.472 | 86 (12) | TNFα | S1 | 0.035 |
| 1 / 4 | 0.308 | 86 (12) | TNFα | S1 | <1E-06 |
| 1 / 6 | 0.215 | 86 (12) | TNFα | S1 | <1E-06 |
| 1 / 8 | 0.339 | 86 (12) | TNFα | S1 | <1E-04 |
| 2 / 6 | 0.455 | 86 (12) | TNFα | S1 | 0.028 |
| 3 / 6 | 0.412 | 86 (12) | TNFα | S1 | 0.004 |
| 0 / 4 | 0.465 | 169 (24) | TNFα | S1 | 0.036 |
| 0 / 6 | 0.215 | 169 (24) | TNFα | S1 | <1E-06 |
| 0 / 8 | 0.233 | 169 (24) | TNFα | S1 | <1E-06 |
| 1 / 6 | 0.240 | 169 (24) | TNFα | S1 | <1E-06 |
| 1 / 8 | 0.260 | 169 (24) | TNFα | S1 | <1E-06 |
| 3 / 6 | 0.350 | 169 (24) | TNFα | S1 | <1E-03 |
| 3 / 8 | 0.380 | 169 (24) | TNFα | S1 | 0.001 |
| 4 / 6 | 0.462 | 169 (24) | TNFα | S1 | 0.022 |
| 0 / 1 | 0.229 | 7 (1) | IFNγ | S2 | 0.006 |
| 0 / 4 | 0.236 | 7 (1) | IFNγ | S2 | <1E-05 |
| 0 / 6 | 0.217 | 7 (1) | IFNγ | S2 | <1E-06 |
| 0 / 8 | 0.229 | 7 (1) | IFNγ | S2 | <1E-06 |
| 1 / 2 | 4.494 | 7 (1) | IFNγ | S2 | 0.013 |
| 2 / 4 | 0.229 | 7 (1) | IFNγ | S2 | <1E-04 |
| 2 / 6 | 0.211 | 7 (1) | IFNγ | S2 | <1E-06 |
| 2 / 8 | 0.222 | 7 (1) | IFNγ | S2 | <1E-06 |
| 3 / 6 | 0.389 | 7 (1) | IFNγ | S2 | 0.007 |
| 3 / 8 | 0.410 | 7 (1) | IFNγ | S2 | 0.011 |
| 3 / 4 | 0.375 | 30 (4) | IFNγ | S2 | 0.041 |
| 1 / 4 | 0.199 | 86 (12) | IFNγ | S2 | <1E-06 |
| 1 / 6 | 0.215 | 86 (12) | IFNγ | S2 | <1E-06 |
| 1 / 8 | 0.265 | 86 (12) | IFNγ | S2 | <1E-04 |
| 0 / 6 | 0.386 | 169 (24) | IFNγ | S2 | 0.039 |
| 1 / 2 | 0.427 | 169 (24) | IFNγ | S2 | 0.032 |
| 1 / 3 | 0.378 | 169 (24) | IFNγ | S2 | <1E-03 |
| 1 / 4 | 0.336 | 169 (24) | IFNγ | S2 | 0.012 |
| 1 / 6 | 0.271 | 169 (24) | IFNγ | S2 | <1E-04 |
| 1 / 8 | 0.385 | 169 (24) | IFNγ | S2 | <1E-03 |
| 0 / 3 | 3.513 | 7 (1) | IL-2 | S2 | 0.002 |
| 1 / 2 | 5.579 | 7 (1) | IL-2 | S2 | 0.013 |
| 1 / 3 | 9.547 | 7 (1) | IL-2 | S2 | <1E-03 |
| 2 / 8 | 0.145 | 7 (1) | IL-2 | S2 | 0.002 |
| 3 / 4 | 0.177 | 7 (1) | IL-2 | S2 | 0.009 |
| 3 / 6 | 0.328 | 7 (1) | IL-2 | S2 | 0.048 |
| 3 / 8 | 0.085 | 7 (1) | IL-2 | S2 | <1E-05 |
| 0 / 2 | 10.533 | 30 (4) | IL-2 | S2 | 0.019 |
| 0 / 3 | 17.037 | 30 (4) | IL-2 | S2 | <1E-03 |
| 0 / 6 | 13.141 | 30 (4) | IL-2 | S2 | 0.002 |
| 1 / 3 | 5.065 | 30 (4) | IL-2 | S2 | <1E-03 |
| 1 / 6 | 3.907 | 30 (4) | IL-2 | S2 | 0.020 |
| 3 / 4 | 0.224 | 30 (4) | IL-2 | S2 | 0.004 |
| 1 / 3 | 1.956 | 169 (24) | IL-2 | S2 | 0.046 |
| 3 / 4 | 0.301 | 169 (24) | IL-2 | S2 | 0.002 |
| 3 / 6 | 0.359 | 169 (24) | IL-2 | S2 | 0.012 |
| 1 / 4 | 0.221 | 86 (12) | TNFα | S2 | <1E-04 |
| 1 / 6 | 0.275 | 86 (12) | TNFα | S2 | 0.005 |
| 1 / 8 | 0.235 | 86 (12) | TNFα | S2 | <1E-03 |
| 3 / 4 | 0.346 | 86 (12) | TNFα | S2 | 0.002 |
| 3 / 8 | 0.368 | 86 (12) | TNFα | S2 | 0.024 |
| 0 / 6 | 0.322 | 169 (24) | TNFα | S2 | 0.008 |
| 1 / 4 | 0.343 | 169 (24) | TNFα | S2 | 0.012 |
| 1 / 6 | 0.280 | 169 (24) | TNFα | S2 | <1E-03 |
| 1 / 8 | 0.386 | 169 (24) | TNFα | S2 | 0.008 |
| 3 / 6 | 0.352 | 169 (24) | TNFα | S2 | 0.007 |

IFNγ. interferon γ; IL-2. interleukin-2; S1. subunit 1; S2. subunit 2. SARS-CoV-2. severe acute respiratory syndrome coronavirus 2; TNFα. tumor necrosis factor α.

^a^Pairwise comparison X/Y refers to the statistical comparison of the fold change in percentage of SARS-CoV-2 spike-specific IFNγ-producing CD8+ T cells at the X-week dosing interval group compared to Y-week dosing interval group.

^b^P-values are based on a 2-sided t test with multivariate-t adjustment. Though all pairwise comparisons were performed. only those that met a significance level of 0.05 are shown.

### Supplementary Table 8. Statistical Comparisons of Anti-PEG Antibodies Before Dose 2 (IgG and IgM) for mRNA-1273 Dosing Intervals Relative to Control.

| **Pairwise comparison^a^** | **Adjusted *P*-value^b^** |
| --- | --- |
| Anti-PEG IgG | |
| 8 / PBS | 0.914 |
| 6 / PBS | 0.635 |
| 4 / PBS | 0.779 |
| 3 / PBS | 0.133 |
| 2 / PBS | 0.012 |
| 1 / PBS | <1E-05 |
| Prime only / PBS | 0.881 |
| Anti-PEG IgM | |
| 8 / PBS | 0.779 |
| 6 / PBS | 0.531 |
| 4 / PBS | 0.743 |
| 3 / PBS | 0.018 |
| 2 / PBS | 0.001 |
| 1 / PBS | <1E-06 |
| Prime only / PBS | 0.829 |

IgG. immunoglobulin G; IgM. immunoglobulin M; mRNA. messenger RNA; PBS. phosphate-buffered saline; PEG. polyethylene glycol.

Comparisons controlled for mRNA-1273 dose level.

^a^Pairwise comparison X/PBS refers to the statistical comparison of the log_2_ fold change in antibody concentration at the X-week dosing interval group compared to the control group.

^b^P-values are from a 2-sided t test with Dunnett’s adjustment.

## 4. Supplementary References

1. Stasinopoulos DM, Rigby RA. Generalized Additive Models for Location Scale and Shape (GAMLSS) in R. Journal of Statistical Software. 2007 12/31;23(7):1 - 46. doi:10.18637/jss.v023.i07.

2. Box GEP, Cox DR. An Analysis of Transformations. Journal of the Royal Statistical Society Series B (Methodological). 1964;26(2):211-252. Full publication date: 1964.

3. Roederer M, Nozzi JL, Nason MC. SPICE: exploration and analysis of post-cytometric complex multivariate datasets. Cytometry A. 2011 Feb;79(2):167-74. Epub 2011/01/26. doi:10.1002/cyto.a.21015. Cited in: Pubmed; PMID 21265010.

4. Dempster AP, Laird NM, Rubin DB. Maximum Likelihood from Incomplete Data via the EM Algorithm. Journal of the Royal Statistical Society Series B (Methodological). 1977;39(1):1-38. Full publication date: 1977.

5. Rigby R, Stasinopoulos, DM. Generalized additive models for location, scale and shape (with discussion). Journal of the Royal Statistical Society: Series C (Applied Statistics) 2005;54(3):507-554 doi:101111/j1467-9876200500510x. 2005.
